# Older adults using social support to improve self-care (OASIS): Adaptation, implementation and feasibility of peer support for older adults with T2D in appalachia: A feasibility study protocol

**DOI:** 10.1371/journal.pone.0300196

**Published:** 2024-03-18

**Authors:** Brittany L. Smalls, Aaron Kruse-Diehr, Courtney L. Ortz, Key Douthitt, Christopher McLouth, Rachel Shelton, Zoe Taylor, Edith Williams

**Affiliations:** 1 Department of Family and Community Medicine, College of Medicine, University of Kentucky, Lexington, KY, United States of America; 2 Department of Biostatistics, College of Public Health, University of Kentucky, Lexington, KY, United States of America; 3 Department of Sociomedical Sciences, Mailman School of Public Health, Columbia University, New York, NY, United States of America; 4 Center for Community Health and Prevention, School of Medicine and Dentistry, University of Rochester, Rochester, NY, United States of America; PLOS: Public Library of Science, UNITED KINGDOM

## Abstract

**Introduction:**

The prevalence of type 2 diabetes (T2D) is 17% higher in rural dwellers compared to their urban counterparts, and it increases with age, with an estimated 25% of older adults (≥ 65 years) diagnosed. Appropriate self-care is necessary for optimal clinical outcomes. Overall, T2D self-care is consistently poor among the general population but is even worse in rural-dwellers and older adults. In rural Kentucky, up to 23% of adults in Appalachian communities have been diagnosed with T2D and, of those, 26.8% are older adults. To attain optimal clinical outcomes, social environmental factors, including social support, are vital when promoting T2D self-care. Specifically, peer support has shown to be efficacious in improving T2D self-care behaviors and clinical and psychosocial outcomes related to T2D; however, literature also suggests self-selected social support can be obstructive when engaging in healthful activities. Currently available evidence-based interventions (EBIs) using peer support have not been used to prioritize older adults, especially those living in rural communities.

**Method:**

To address this gap, we conducted formative research with stakeholders, and collaboratively identified an acceptable and feasible peer support EBI—peer health coaching (PHC)—that has resulted in improved clinical and psychosocial T2D-related outcomes among participants who did not reside in rural communities nor were ≥65 years. The goal of the proposed study is to use a 2x2 factorial design to test the adapted PHC components and determine their preliminary effectiveness to promote self-care behaviors and improve glycemic control among older adults living in Appalachian Kentucky. Testing the PHC components of the peer support intervention will be instrumental in promoting care for older adults in Appalachia, as it will allow for a larger scale intervention, which if effective, could be disseminated to community partners in Appalachia.

**Trial registration:**

This study was registered at www.clinicaltrials.gov (NCT06003634) in August 2023.

## Introduction

According to the Administration for Community Living [1], 16% (54.1 million) of the United States population is comprised of people aged ≥ 65 years, a percentage poised to rise to 21.6% (80.8 million) by 2040. In Kentucky, older adults account for an estimated 19% of the population, reflective of a 32% increase between 2009 and 2019. The rising number of older adults coincides with increased prevalence of chronic illness, including T2D. At present, an estimated 29% of older adults (≥ 65 years) have diagnosed or undiagnosed T2D [1]. Moreover, older adults living with T2D also have two times the annual healthcare expenditures as their younger counterparts—$13,239 versus $6,675, respectively [1].

Appropriate self-care behaviors are necessary for optimal clinical outcomes in T2D [2–4], accounting for 90% of variance in glycemic control [5]. Older adults living with T2D have more difficulty adhering to self-care regimens, and social environmental factors account for up to 85% of their self-care nonadherence compared to younger adults living with T2D [4, 6–10]. Notable social environmental factors include self-efficacy, distress, and lack of social support [11–14]. Specifically, social support plays a significant role in older adults’ T2D self-care and is thus an important social environmental factor to target in people with T2D [15]. Older rural-dwelling adults diagnosed with chronic disease have worse social function and emotional well-being than older adults living in urban areas [16]. Although social support has been shown to have a significant, independent relationship with T2D self-care, the extent and type of social support that may be available in rural communities might either positively or negatively impact older rural-dwelling adults’ self-care. The type and quality of social support can either reinforce or mitigate structural and psychosocial health determinants—such as healthcare mistrust, cultural health beliefs, and access to healthcare—that can influence self-care behaviors and beliefs [17–22]. Nevertheless, there remains a body of literature that substantiates the influence of social support on T2D self-care [15, 23] as well as the disproportionate burden of T2D in older adults [24].

In addition, frequency of desired social contact is also important for older adults. Previous research shows that more frequent social contact can mitigate depressive symptoms, frailty, and cognitive decline [25–27]. Frequencies of contact ranging from daily or weekly to monthly are each associated with health and wellbeing in older adults [25–27]. When designing and administering social support interventions in older adults, ensuring the appropriate type of support and determining the necessary contact frequency are both important considerations to providing appropriate social support for T2D self-care.

This study focuses on peer support. Peer support, wherein a person receives “support from a person who has experiential knowledge of a specific behavior or stressors and similar characteristics,” provides a mechanism for creating a social network that augments existing social supports [28]. In a systematic review of 25 randomized trials assessing structured peer support interventions for individuals with T2D, there were significant differences in HbA1c, blood pressure, cholesterol, and symptoms of hypo- or hyperglycemia between peer support and other study arms [29], highlighting the utility of structured peer support as an effective means to improving health behaviors and outcomes in low income and minority groups [30–33]. Because rural Appalachia Kentucky is characterized by close knit communities, the use of structured social support interventions, such as peer support, to promote desired health behaviors might be both feasible and acceptable to community members.

The present paper describes the research protocol for a feasibility study entitled, *Older Adults Using Social Support to Improve Self-Care (OASIS)*: *Adaptation*, *Implementation and Feasibility of Peer Support for Older Adults with T2D in Appalachia*. The goal of OASIS is to test specific components of a peer support intervention, PHC, and determine components that are most effective at promoting self-care behaviors and improving glycemic control among older adults living in Appalachian Kentucky.

## Methods

### Study aims

This study is guided by the following two aims:

#### 1. Use a 2x2 factorial design to determine which EBI components are associated with improved T2D-related outcomes in older adults living in Appalachia Kentucky

The independent variables for the factorial design are (1) how the peer coach is selected (self-selected by participant vs matched) and (2) frequency of contact (once per week vs every 2 weeks). We will evaluate effectiveness of each group using hemoglobin A1c (HbA1c) as the primary outcome while also evaluating self-care activities and T2D-related psychosocial factors as secondary outcomes

#### 2. Evaluate the pragmatic implementability of the adapted EBI

Guided by the Practical, Robust Implementation and Sustainability Model (PRISM), a framework that incorporates RE-AIM dimensions (i.e., reach, effectiveness, adoption, implementation, and maintenance). Interviews will be conducted at three timepoints: (1) at beginning of implementation, (2) at implementation midpoint, and (3) upon conclusion. We will collect both qualitative and quantitative data to assess PRISM domains and give context to both anticipated and unanticipated EBI adaptations. We will use an iterative approach to RE-AIM to collaboratively monitor RE-AIM dimensions, guide any needed mid-course adjustments, and evaluate implementation outcomes. Finally, feasibility, appropriateness, and acceptability of the EBI will be assessed via a validated 12-item questionnaire.

### Data safety, monitoring and management

The co-principal investigators (PIs; BLS and AKD) will ensure that all research personnel have undergone Collaborative Institutional Training Initiative (CITI) training for conducting human subjects research and gaining access to study data. They will monitor the study to ensure that all potential risks to participants remain minimal. In addition, all research personnel will be provided with a standard operating procedure manual that outlines recruitment strategies; the informed consent process including study procedures, contact information for the University of Kentucky Office of Research Integrity as well as the principal investigator, study timeline, and an appendix of required data collection documents; and a copy of the most recent consent forms and institutional review board study approval letter. The co-PIs will also invite the Office of Research Integrity to conduct mock audits on data collection and de-identification, encryption of audio files, and informed consent documentation to ensure research participants are properly protected.

#### Confidentiality

All data will be de-identified and stored on a password-protected computer at the University of Kentucky, including health information. In order to track participation over time, we will use an enrollment log that includes participants’ study IDs but will only use study IDs (e.g., de-identify) when entering data into REDCap. Only authorized individuals will have access to study data. Data and electronic records will be kept for a minimum of six years post-study closure. All precautions will be taken to keep participants safe. To secure against a potential breach of confidentiality, all study files will be deidentified and kept on a password-protected secure server at the University of Kentucky.

If there is any suspicion that a subject does not want to continue participating in the study, they will be withdrawn from the study. If a subject indicates that their participation in the study is causing distress, one of the co-PIs will telephone the participant within 24 hours to see how he/she is managing and may suggest he/she contact his/her primary care provider. In the event the subject does not have a primary care provider, one of the co-PIs will offer the subject a list of appropriate referral sources if he/she wishes. In addition, to avoid future incidents, each case of possible distress will be reviewed with the co-PIs and appropriate members of the investigative team, including review of study materials and responses to distressed participants. This review will evaluate whether any aspect of the study need requires modification.

#### Ethics approval and consent to participate

The study was initially approved by the Institutional Review Board (IRB) of the University of Kentucky on March 3, 2023 (protocol # 83904). Research staff members will obtain written informed consent for all participants meeting inclusion criteria prior to study enrollment. Recruitment for this study began October 1, 2023 and will conclude March 30, 2026.

#### Plans to promote retention and complete follow-up

If a participant chooses to leave the study early, data collected until that point will remain in the study database and may not be removed. The intervention will no longer be provided to participants who are removed from this study, and participants will not be compensated for any sessions completed after being removed.

#### Protocol nonadherence and amendments

Participants will not be removed from the study for nonadherence to the study protocol or for missing data. Protocol amendments will be communicated to funding agencies, trial participants, IRB officials, and trial registries.

### Stakeholders

#### Stakeholders eligibility

- Age ≥ 18.

#### Recruitment of stakeholders

This is a purposive sample of stakeholders (e.g., community leaders, older adults with T2D, representatives from Barren River Area Development District (BRADD) organizations [Aging on Aging case worker, Community Action]), who will provide feedback, ensure fidelity, and acceptability of the final evidence-based intervention.

#### Pre-intervention

Prior to the start of the intervention, the research team will obtain informed consent and will conduct up to three stakeholder meetings with the following goals: (1) present the final adapted version of the peer health coaching EBI to identify any possible final adaptations to promote pragmatic implementability; (2) finalize operationalization of RE-AIM dimensions based on shared implementation goals; and (3) identify potential facilitators and barriers that might influence implementation. To achieve the third goal, we will use an interview guide tested in previous applications of PRISM [34] across health systems to analyze PRISM contextual determinants, such as external environment, implementation and sustainability infrastructure, and characteristics of participants of the EBI and of the EBI itself. See S1 Fig for study activities involving stakeholders, peer coaches, and peer participants.

### Peer health coaches

#### Peer health coach eligibility

Coaches will be required to:

- have a HbA1c less than 7.5% at time of enrollment
- age greater than or equal to 55
- residence in Appalachia Kentucky.

#### Recruitment of peer health coaches

Potential peer coaches will be invited from community centers and senior citizen centers in BRADD. We will mail out recruitment letters that will explain the study and provide contact information for participants to call if they are interested in participating. The same peer health coaches will be involved in all three waves of participant recruitment.

#### Screening of peer health coaches

In addition to meeting the eligibility criteria listed above, peer health coaches will complete an interview with the PI, where the PI will assess peer coach psychological status using the Wallston General Perceived Competence Scale, Campbell Personal Competence Index, Carkhuff Communication and Discrimination Skills Inventories, and Applied Knowledge Assessment (AKA) Scale [35].

#### Pre-intervention training of peer health coaches

The research team will obtain consent from peer coaches. Each potential peer coach will receive 2–3 hours of training. They will complete structured curriculum modules on working collaboratively with study participants, basics of diabetes including self-care activities, knowledge of diabetes medications, recognizing medical "red flags" (e.g., symptoms of hypoglycemia), navigating the clinic, and assessing community resources. The peer coaches will also have training in informal skills development including active listening, non-judgmental communication, and positive social and emotional support. The co-PIs will determine competence, maturity, emotional stability, and verbal communication skills after overall assessment during the screening interview and training. Only coaches who pass a written and oral examination are included in the study.

### Peer participants

#### Sample size

From a statistical perspective, the goal of this pilot and feasibility study is to obtain precise estimates of outcome variability and preliminary effect sizes for each factor (peer support intervention, contact frequency) that can be used to power a large-scale clinical trial. Empirical work by Whitehead and colleagues [36] provides guidance on sample size requirements for pilot studies to accurately estimate the needed size of future trials. For a main trial that aims for 90% power with a two-sided α = .05, a pilot sample size per arm of 25 would be needed in anticipation of a small standardized effect size (e.g., Cohen’s f = 0.1). This study is interested not only in main effects, but also an interaction between peer support intervention and contact frequency. Given that interactions are notoriously underpowered [37]—and given anticipated participant attrition—the decision was made to recruit 19 participants per condition for a total sample size of 76. With this sample size, the detectable effect size for a between-group difference in the primary outcome is Cohen’s f = 0.33 at 80% power with a two-sided α = .05.

### Peer participant eligibility criteria

Eligibility criteria for peer participants include:

- confirmed diagnosis of T2D via point-of-care HbA1c assessment;
- residence in Appalachia Kentucky;
- HbA1c ≥ 7.5% in the previous 6 months;
- age ≥ 55 years.

#### Recruitment of peer participants

Peer participants will be recruited from community-based organizations located in the BRADD. Recruitment and enrollment will occur in 3 waves. Within each wave, each coach will be assigned all of their peer participants at one time to ensure that intervention activities occur within the same time period.

#### Screening of peer participants

All individuals who are determined to be eligible for the study will then be screened for cognitive impairment using the Montreal Cognitive Assessment-Basic (MoCA-B) [38] to ensure that study participants have the capacity to engage in study activities.

#### Random assignment of peer participants to intervention condition

Participants will be consented, enrolled and randomized into one of four groups. During the consenting process, it will be explained that participants can withdraw at any time. Participant can be randomized into one of the following groups: (1) self-select mentor, once per week contact; (2) self-select mentor, every two weeks contact; (3) matched with mentor, once per week contact; or (4) matched with mentor, every two weeks contact. For participants who are randomized into a group where they are matched with a peer health coach, matching areas include similarity of life stage (e.g., age), county of residence, and duration of disease diagnosis. However, the primary matching area for this study will be county of residence. This decision was informed by key informants who highlighted that Appalachians’ sense of identity is closely linked with the county in which they reside and that there will be an additional level of trust and comfort (or lack thereof) based on where their peer resides. For participants who are randomized into the group where they are able to self-select their peer coach, they will be provided with profiles of eligible coaches. These profiles will include age, sex, marital status, where they reside (county and town), duration of diabetes diagnosis, current HbA1c, and hobbies. Peer participants will be able to rank their top three coaches. The study team will try to match participants with their preferred coach. However, once a coach has been assigned three peer participants, they will be removed from the pool of coaches to choose from.

### Introductory session for peer coaches and participants

Peer participants and coaches will attend an introductory session together, during which the coaching process will be discussed, including time commitment, roles, responsibilities, benefits, and ground rules. At that time, peer participants who are randomized to self-select their coaches will have the opportunity to review peer coach profiles and rank their top three coaches to be matched. Once coaches and peers have been selected, the quads (coach with three peers) will have the opportunity to ask questions and make informed decisions about their ability to fully participate in the intervention. If face-to-face meetings are not possible for all members of the “quad,” phone or a form of video meeting will be attempted. At this time, the research coordinator will collect demographics, validated questionnaires, and baseline point-of-care clinical outcomes (HbA1c via fingerstick). This introductory meeting as well as the peer coach-peer participant meetings during the six months of intervention can be in-person in community settings or via Zoom, depending upon participants/coach preference.

### Intervention for peer coaches and participants

During the intervention, peer coaches interact with the peer participant for six months, with the frequency of interactions based on whichever group the peer participant was randomly assigned to as previously described. It is also optional for the peer coach to accompany the participant to at least one clinic visit. Topics to be discussed include current and target clinical goals for HbA1C, cholesterol, blood pressure, self-care activities, managing stress, and the SMART objectives. Coach-peer interactions will be documented with the following information: date, type of encounter (phone, in-person), duration, and topics discussed. Coaches will attend a monthly meeting to reinforce diabetes knowledge and communication skills. This meeting will be an open discussion based on their experiences interfacing with participants. Zoom will be used for the monthly meetings. Peer participants will receive a link to a brief REDCap survey by email every two weeks. If internet access is limited, we will send a hardcopy to the participant with a self-addressed stamped envelope. This survey will assess the frequency and duration of calls, other interactions with their peer, and the specific content that was covered. These self-report assessments will be used to track the effectiveness of the intervention.

### Mid-intervention

At mid-intervention, we will again convene stakeholders for two additional meetings with three primary goals: (1) assess progress on RE-AIM dimensions; (2) select RE-AIM dimensions that require additional attention, if necessary; and (3) give context to understand the reasoning behind any differences that might exist between pre-implementation (i.e., anticipated) and midstream (i.e., unanticipated) adaptations. To achieve the first two goals, we will use an iterative approach to RE-AIM, wherein at the first meeting, team members will be reminded of RE-AIM dimensions selected at project start-up and then asked to confidentially rate the importance and progress of each RE-AIM dimension thus far on a 5-point Likert scale with options to provide qualitative explanatory feedback for each rating. At the second meeting, we will present de-identified results from the survey and then engage in subsequent brainstorming/goal setting to determine best approaches, including possible midcourse EBI adaptations, to improve data collection for RE-AIM dimensions deemed most in need of increased attention. To meet the third goal, we will use a PRISM-based survey [39] and the same interview guide used at project start-up to give context to determinants of midcourse progress (including any identified adaptations).

### Post-intervention

#### Peer coaches and participants

At the end of the intervention, the research coordinator will collect HbA1C via fingerstick and the validated questionnaire data described in data collection from peer coaches and peer participants. Peer participants will then provide HbA1C levels and validated questionnaires again at 3- and 6-months post intervention.

#### Stakeholders

Finally, immediately upon program conclusion, we will convene a final team meeting with stakeholders, as well as a sample of randomly selected peer health coaches from each of the four factorial conditions, to collect RE-AIM outcomes data and conduct summative interviews with the same PRISM survey and qualitative interview guide as used at project onset and midpoint. Additionally, we will ask stakeholders and selected peer health coaches to complete a psychometrically validated brief 12-item survey [40] on perceptions of feasibility, acceptability, and appropriateness of both the overall EBI and each of the selected strategies (i.e., each component in the 2x2 factorial design); we will also randomly select participants from each condition to complete the same survey and complete exit interviews to explore perceived and actual factors underscoring participant satisfaction with the EBI and its components.

### Study measurements and outcomes

Stakeholder’s data will be collected via semi-structured interviews pre-intervention, mid-intervention and post-intervention. Survey data will also be collected from stakeholders’ post-intervention. Peer coaches and peer participants will provide HbA1C levels via blood draw. Peer coaches will complete blood draws and questionnaire data at baseline and end of intervention across all three waves, whereas peer participants will complete blood draws and questionnaire data at baseline, end of intervention (6 months), 9 months and 12 months (see Table 1).

**Table 1:** Data collection measure instruments.

Measure	Method
**Primary Outcomes**	
Hemoglobin A1C	This will be collected via point-of-care assessment.
Medication Adherence	The Brooks Medication Adherence Scale will be used to assess medication adherence [41].
Quality of Life	The EuroQol-5D (EQ-5D) is a validated measure used to measure quality of life [42].
Diabetes-related emotional distress	The Problem Areas in Diabetes Survey (PAID) will assess diabetes-related emotional distress among adults [43].
Perceived Social Support	The Medical Outcomes Study (MOS) Social Support Survey will be used to assess perceived social support [44].
Problem-Solving Skills	The Diabetes Problem-Solving Inventory (DPSI) will assess how individuals living with diabetes cope with challenges of T2D-related self-care [45].
Depression	The Geriatric Depression Scale will be used to measure depression [46].
Self-Efficacy	The Diabetes Empowerment Scale measures diabetes-related psychosocial self-efficacy [47].
Diabetes Knowledge	The Diabetes Knowledge Questionnaire (DKQ) measures diabetes knowledge intervention [48].
Self-Care	The Diabetes Self-Management Questionnaire will assess self-care activity associated with glycemic control [49].
**Secondary Outcomes**	
Refine proposed operationalizations of RE-AIM dimensions	Qualitative interviews
Assess RE-AIM dimensions	Qualitative interviews
RE-AIM outcomes	Qualitative interviews
PRISM survey and interview guide	A PRISM-based survey [40] and an interview guide tested in previous applications of PRISM [34] across health systems to analyze PRISM contextual determinants
Feasibility, acceptability, and appropriateness survey	A psychometrically validated brief 12-item survey [40]
**Covariates**	
Personal characteristics (Age, Sex, Race/Ethnicity, Marital Status, County of Residence, Income)	All participants will provide answers to these personal characteristics
Physical Function	Lawton’s Instrumental Activities for Daily Living Scale will assess physical function [50].
Frailty	This will be assessed using a validated 4-item assessment that characterizes frailty by exhaustion, low physical activity, weakness, and low body mass index [51, 52].

#### Study measurements

The study measurements collected in the intervention include peer coach and participant interactions. Specifically, coach-peer interactions will be documented with the following: date, type of encounter (phone, in-person), duration, and topics discussed. Topics to be discussed include current and target clinical goals for A1c, cholesterol, blood pressure, self-care activities, managing stress, and SMART objectives. Self-report assessments will be used to track the effectiveness of the intervention.

#### Primary study outcomes

The outcomes of this study will be assessed against the objective of implementing a peer support intervention in Appalachia to improve the management of T2D in older adults.

(1) To use a 2x2 factorial design to determine which EBI components are associated with improved T2D-related outcomes in older adults living in Appalachia Kentucky by evaluating:

a. Peer coach selection (matched vs. self-selected) and frequency of contact (once per week vs. every 2 weeks) on
  - HbA1c, which will be collected via point-of-care assessment.
  - T2D-related psychosocial factors (see Table 1).

#### Secondary study outcomes

(1) Evaluate the pragmatic implementability of the adapted EBI
  - To better understand implementability of the overall EBI, as well as each of the four strategies tested in the 2x2 factorial design, we will assess contextual factors that influence EBI adoption as well as implementation outcomes using the PRISM [53], a framework that incorporates RE-AIM dimensions. We will use a novel iterative approach to RE-AIM [54], wherein team members (both researchers and implementers) will collectively refine proposed operationalizations of RE-AIM dimensions and then, at project mid-point, identify RE-AIM dimensions needing added attention and set goals and strategies to improve progress on them, including potentially adapting or rejecting and replacing indicators to better capture necessary outcomes data. This iterative use of RE-AIM will ensure that any midcourse corrections are informed by pragmatic need and are documented accordingly.

## Data analyses

### Descriptive analysis

Descriptive data in the form of means (standard deviations) for interval-level variables and frequencies (percentages) for qualitative variables will be presented for the entire population and by trial arm. Prior to conducting and interpreting the main analyses, the distribution of all outcome variables will be examined to ensure the tenability of parametric assumptions.

### Analytic plan for aim 1

The repeated measures, factorial design will be analyzed using longitudinal linear mixed models. Linear mixed effects models are an extension of the general linear model and are suitable when the independence of observations assumption is violated, which occurs in this study when individuals are measured repeatedly over time. Additionally, these models have the benefit over a repeated measures ANOVA of utilizing all data from participants who were measured on some, but not necessarily all of the measurement occasions. The main outcome for this aim is HbA1c levels at the first post-intervention measurement occasion. Fixed effects of interest will include measurement occasion, peer support type, and contact frequency. Preliminary models will include all two-way interactions, and non-significant higher-order interactions will be removed to arrive at the most parsimonious model. The model will incorporate random effects to account for repeated measures on individuals using an autoregressive covariance matrix. Due to randomization, we do not anticipate any differences in covariates between groups; however, we will evaluate differences by main effects and adjust for them if necessary. Additional analyses related to Aim 1 will look at between-group differences in psychosocial factors over time.

#### Exploratory analyses

Exploratory analyses seek to address the concern that the number of peers assigned to a peer coach may impact their ability to successfully coach individuals with T2D and may have a potentially deleterious impact on their own T2D self-care. This hypothesis will again be tested using longitudinal linear mixed models. HbA1c levels over time (for both peers and peer coaches) will be modeled as a function of the number of concurrent peers a peer coach has. This will help inform future studies of the ideal number of peers a peer coach has at any given time.

#### Assessing implementation

For all PRISM-guided interviews, two members of the research team with academic training and pragmatic experience in implementation science as well as substantial qualitative research experience will independently code the data and map responses to relevant PRISM domains, as outlined in previous applications of PRISM [34]. Should coding conflicts arise, a skilled implementation scientist with ample research using PRISM/RE-AIM will mediate and help build coding consensus. Data from midpoint iterative RE-AIM planning will be used primarily as quality improvement and will be presented to members of the implementation and research teams using descriptive statistics, graphs, charts, and de-identified thematic analysis of any explanatory feedback. Finally, for the 12-item feasibility, acceptability and appropriateness survey, scores for each of the three scales will be summed and averaged, with higher scores indicating more favorable implementation outcomes. These data will provide added context to which factorial condition was perceived by both implementers and participants as most favorable. Collectively, these data will identify necessary implementation strategies, as well as facilitators and barriers to implementation, to guide future scale-up and scale-out activities throughout rural Appalachia.

## Discussion

Peer health coaching is an approach that relies on social relationships and has resulted in improved outcomes in participants with T2D. In studies of predominantly low-income and minority populations, peer health coaches have been shown to help improve medication adherence and blood glucose monitoring [55–61]. Peer health coaches are usually individuals who have successfully coped with a similar condition as their peer [28]. In peer health coaching interventions, coaches receive training focused on communication skills, including empathetic listening, helping peers clarify life goals, and problem solving with the aim of having the coach support their peer [62]. For the current study, we have chosen a peer coach EBI that showed an HbA1c reduction of 1.07% among peer participants [63].

We acknowledge the following potential challenges with this proposal. First, we may encounter challenges with recruitment and retainment of older adults in rural communities; however, we involved key stakeholders in the development of this study and have included their recommendations in our recruitment efforts along with previously successful recruitment strategies. Second, we acknowledge that older adults may find the use of REDCap links to complete data collection at baseline, 6 months (end of intervention), and 3- and 6-months post-intervention challenging; however, these potential challenges are integral to the evaluation and implementation of the intervention. Lastly, we recognize the lack of a usual care arm may be considered a weakness of our study design and/or a challenge to the interpretation of our findings. Still, we opted not to have a usual care arm because of the dire circumstances of those diagnosed with T2D living in Appalachia Kentucky (e.g., high prevalence, disease severity, lack of resources). In addition, the goal of this pilot and feasibility study is to identify efficacious components to refine a peer support EBI targeting older adults in rural Appalachia. The factorial design provides the scientific rigor to accomplish this goal without the use of a control or usual care group.

The findings from this study will be used to test the effectiveness of the refined intervention in a larger, adequately powered study. Data collected on implementation science components, feasibility, and acceptability will be used to scale up the peer health coach intervention. Lastly, the subsequent study will assess the healthcare utilization/cost effectiveness component of the Main Effects of Social Support Model of the large-scale intervention (e.g., economic evaluation of cost, implementation specific cost assessment) as well as considerations for sustainability. In conclusion, by delivering a tailored, evidence-based peer support intervention (i.e., peer health coaching), we will gain valuable insight in how to leverage existing social cohesion and social support within rural Appalachian Kentucky to promote healthy behaviors and improve T2D related clinical outcomes.

## Supporting information

S1 Fig Flowchart of OASIS study activities. (TIF)

S1 File (DOC)

S2 File (PDF)

S3 File (PDF)

## Abbreviations

T2D: Type 2 Diabetes
EBI: Evidence-Based Intervention
PHC: Peer Health Coaching
PRISM: Practical Robust Implementation and Sustainability Model
BRADD: Barren River Area Development District

## References

[1] Administration for Community Living. 2020 Profile of Older Americans, 2021.

[2] Toobert DJ, Hampson SE, Glasgow RE. The summary of diabetes self-care activities measure: results from 7 studies and a revised scale. *Diabetes Care*. 2000; 23(7): 943–50. doi: 10.2337/diacare.23.7.943 10895844

[3] Shrivastava SR, Shrivastava PS, Ramasamy J. Role of self-care in management of diabetes mellitus. *J Diabetes Metab Disord*. 2013;12(1):14. Published 2013 Mar 5. doi: 10.1186/2251-6581-12-14 23497559 PMC3599009

[4] Asante E. Interventions to promote treatment adherence in type 2 diabetes mellitus. *Br J Community Nurs*. 2013;18(6):267–274. doi: 10.12968/bjcn.2013.18.6.267 24046923

[5] Tuerk PW, Mueller M, Egede LE. Estimating physician effects on glycemic control in the treatment of diabetes: methods, effects sizes, and implications for treatment policy. *Diabetes Care*. 2008; 31(5): 869–73. doi: 10.2337/dc07-1662 18285552

[6] Billimek J, Sorkin DH. Self-reported neighborhood safety and nonadherence to treatment regimens among patients with type 2 diabetes. *J Gen Intern Med*. 2012; 27(3): 292–6. doi: 10.1007/s11606-011-1882-7 21935749 PMC3286552

[7] Gary TL, Safford MM, Gerzoff RB, et al. Perception of neighborhood problems, health behaviors, and diabetes outcomes among adults with diabetes in managed care: the Translating Research into Action for Diabetes (TRIAD) study. *Diabetes Care*. 2008; 31(2): 273–8. doi: 10.2337/dc07-1111 18000180

[8] Paes AH, Bakker A, Soe-Agnie CJ. Impact of dosage frequency on patient compliance. *Diabetes Care*. 1997; 20(10): 1512–7. doi: 10.2337/diacare.20.10.1512 9314626

[9] Cramer JA. A systematic review of adherence with medications for diabetes. *Diabetes Care*. 2004; 27(5): 1218–24. doi: 10.2337/diacare.27.5.1218 15111553

[10] Yusuff KB, Obe O, Joseph BY. Adherence to anti-diabetic drug therapy and self management practices among type-2 diabetics in Nigeria. *Pharm World Sci*. 2008; 30(6): 876–83. doi: 10.1007/s11096-008-9243-2 18784982

[11] Schoenberg NE, Leach C, Edwards W. "It’s a toss up between my hearing, my heart, and my hip": prioritizing and accommodating multiple morbidities by vulnerable older adults. *J Health Care Poor Underserved*. 2009;20(1):134–151. doi: 10.1353/hpu.0.0115 19202253 PMC3025858

[12] Schoenberg NE, Drungle SC. Barriers to non-insulin dependent diabetes mellitus (NIDDM) self-care practices among older women. *J Aging Health*. 2001;13(4):443–466. doi: 10.1177/089826430101300401 11813736

[13] Nicklett EJ, Heisler ME, Spencer MS, Rosland AM. Direct social support and long-term health among middle-aged and older adults with type 2 diabetes mellitus [published correction appears in J Gerontol B Psychol Sci Soc Sci. 2014 Mar;69(2):336]. *J Gerontol B Psychol Sci Soc Sci*. 2013;68(6):933–943. doi: 10.1093/geronb/gbt100 24150176 PMC3805290

[14] Mayer-Davis EJ, D’Antonio AM, Smith SM, et al. Pounds off with empowerment (POWER): a clinical trial of weight management strategies for black and white adults with diabetes who live in medically underserved rural communities. *Am J Public Health*. 2004;94(10):1736–1742. doi: 10.2105/ajph.94.10.1736 15451743 PMC1448527

[15] Strom JL, Egede LE. The impact of social support on outcomes in adult patients with type 2 diabetes: a systematic review. *Curr Diab Rep*. 2012;12(6):769–781. doi: 10.1007/s11892-012-0317-0 22949135 PMC3490012

[16] Baernholdt M, Yan G, Hinton I, Rose K, Mattos M. Quality of life in rural and urban adults 65 years and older: findings from the National Health and Nutrition Examination survey. *J Rural Health*. 2012;28(4):339–347. doi: 10.1111/j.1748-0361.2011.00403.x 23083080 PMC3615459

[17] Carter-Edwards L, Skelly AH, Cagle CS, Appel SJ. "They care but don’t understand": family support of African American women with type 2 diabetes. *Diabetes Educ*. 2004;30(3):493–501. doi: 10.1177/014572170403000321 15208847

[18] Glasgow RE, Toobert DJ. Social environment and regimen adherence among type II diabetic patients. *Diabetes Care*. 1988; 11(5): 377–86. doi: 10.2337/diacare.11.5.377 3391088

[19] Henry SL, Rook KS, Stephens MA, Franks MM. Spousal undermining of older diabetic patients’ disease management. *J Health Psychol*. 2013;18(12):1550–1561. doi: 10.1177/1359105312465913 23325381 PMC4506743

[20] Mayberry LS, Berg CA, Greevy RA Jr., Wallston KA. Assessing helpful and harmful family and friend involvement in adults’ type 2 diabetes self-management. Patient Educ Couns. 2019; 102(7): 1380–8. doi: 10.1016/j.pec.2019.02.027 30922622 PMC6546510

[21] Mayberry LS, Osborn CY. Family support, medication adherence, and glycemic control among adults with type 2 diabetes. *Diabetes Care*. 2012; 35(6): 1239–45. doi: 10.2337/dc11-2103 22538012 PMC3357235

[22] Mayberry LS, Rothman RL, Osborn CY. Family members’ obstructive behaviors appear to be more harmful among adults with type 2 diabetes and limited health literacy. *J Health Commun*. 2014;19 Suppl 2(0 2):132–143. doi: 10.1080/10810730.2014.938840 25315589 PMC4199389

[23] Gallant MP. The influence of social support on chronic illness self-management: a review and directions for research. *Health Educ Behav*. 2003;30(2):170–195. doi: 10.1177/1090198102251030 12693522

[24] Kirkman MS, Briscoe VJ, Clark N, et al. Diabetes in older adults. *Diabetes Care*. 2012; 35(12): 2650–64. doi: 10.2337/dc12-1801 23100048 PMC3507610

[25] Chon D, Lee Y, Kim J, Lee KE. The Association between Frequency of Social Contact and Frailty in Older People: Korean Frailty and Aging Cohort Study (KFACS). *J Korean Med Sci*. 2018;33(51):e332. Published 2018 Dec 3. doi: 10.3346/jkms.2018.33.e332 30546284 PMC6291405

[26] Teo AR, Choi H, Andrea SB, et al. Does Mode of Contact with Different Types of Social Relationships Predict Depression in Older Adults? Evidence from a Nationally Representative Survey. *J Am Geriatr Soc*. 2015;63(10):2014–2022. doi: 10.1111/jgs.13667 26437566 PMC5527991

[27] Schwartz E, Khalaila R, Litwin H. Contact frequency and cognitive health among older adults in Israel. *Aging Ment Health*. 2019;23(8):1008–1016. doi: 10.1080/13607863.2018.1459472 29723058 PMC6215531

[28] Brownson CA, Heisler M. The role of peer support in diabetes care and self-management. *Patient*. 2009;2(1):5–17. doi: 10.2165/01312067-200902010-00002 22273055

[29] Dale JR, Williams SM, Bowyer V. What is the effect of peer support on diabetes outcomes in adults? A systematic review. *Diabet Med*. 2012;29(11):1361–1377. doi: 10.1111/j.1464-5491.2012.03749.x 22804713

[30] Armstrong K, McMurphy S, Dean LT, et al. Differences in the patterns of health care system distrust between blacks and whites. *J Gen Intern Med*. 2008;23(6):827–833. doi: 10.1007/s11606-008-0561-9 18299939 PMC2517897

[31] Ghahramani N. Potential impact of peer mentoring on treatment choice in patients with chronic kidney disease: a review. *Arch Iran Med*. 2015;18(4):239–243. 25841945

[32] Feldman CH, Bermas BL, Zibit M, et al. Designing an intervention for women with systemic lupus erythematosus from medically underserved areas to improve care: a qualitative study. *Lupus*. 2013; 22(1): 52–62. doi: 10.1177/0961203312463979 23087258 PMC3543784

[33] Sokol R, Fisher E. Peer Support for the Hardly Reached: A Systematic Review. *Am J Public Health*. 2016;106(7):e1–e8. doi: 10.2105/AJPH.2016.303180 27196645 PMC4984766

[34] McCreight MS, Rabin BA, Glasgow RE, et al. Using the Practical, Robust Implementation and Sustainability Model (PRISM) to qualitatively assess multilevel contextual factors to help plan, implement, evaluate, and disseminate health services programs. *Transl Behav Med*. 2019;9(6):1002–1011. doi: 10.1093/tbm/ibz085 31170296

[35] Peterson MG, Horton R, Engelhard E, Lockshin MD, Abramson T. Effect of counselor training on skills development and psychosocial status of volunteers with systemic lupus erythematosus. *Arthritis Care Res*. 1993;6(1):38–44. doi: 10.1002/art.1790060108 8443256

[36] Whitehead AL, Julious SA, Cooper CL, Campbell MJ. Estimating the sample size for a pilot randomised trial to minimise the overall trial sample size for the external pilot and main trial for a continuous outcome variable. *Stat Methods Med Res*. 2016;25(3):1057–1073. doi: 10.1177/0962280215588241 26092476 PMC4876429

[37] Maxwell SE. The persistence of underpowered studies in psychological research: causes, consequences, and remedies. *Psychol Methods*. 2004;9(2):147–163. doi: 10.1037/1082-989X.9.2.147 15137886

[38] Julayanont P, Tangwongchai S, Hemrungrojn S, et al. The Montreal Cognitive Assessment-Basic: A Screening Tool for Mild Cognitive Impairment in Illiterate and Low-Educated Elderly Adults. *J Am Geriatr Soc*. 2015;63(12):2550–2554. doi: 10.1111/jgs.13820 26648041

[39] Pittman JOE, Lindamer L, Afari N, et al. Implementing eScreening for suicide prevention in VA post-9/11 transition programs using a stepped-wedge, mixed-method, hybrid type-II implementation trial: a study protocol. *Implement Sci Commun*. 2021;2(1):46. Published 2021 Apr 29. doi: 10.1186/s43058-021-00142-9 33926577 PMC8082763

[40] Weiner BJ, Lewis CC, Stanick C, et al. Psychometric assessment of three newly developed implementation outcome measures. *Implement Sci*. 2017;12(1):108. Published 2017 Aug 29. doi: 10.1186/s13012-017-0635-3 28851459 PMC5576104

[41] Brooks CM, Richards JM, Kohler CL, et al. Assessing Adherence to Asthma Medication and Inhaler Regimens: A Psychometric Analysis of Adult Self-Report Scales. *Med Care*. 1994; 32(3): 298–307. doi: 10.1097/00005650-199403000-00008 8145604

[42] Rabin R, de Charro F. EQ-5D: a measure of health status from the EuroQol Group. *Ann Med*. 2001;33(5):337–343. doi: 10.3109/07853890109002087 11491192

[43] Polonsky WH, Anderson BJ, Lohrer PA, et al. Assessment of diabetes-related distress. *Diabetes Care*. 1995; 18(6): 754–60. doi: 10.2337/diacare.18.6.754 7555499

[44] Sherbourne CD, Stewart AL. The MOS social support survey. *Soc Sci Med*. 1991; 32(6): 705–14. doi: 10.1016/0277-9536(91)90150-b 2035047

[45] Glasgow RE, Toobert DJ, Barrera M Jr, Strycker LA. Assessment of problem-solving: a key to successful diabetes self-management. *J Behav Med*. 2004;27(5):477–490. doi: 10.1023/b:jobm.0000047611.81027.71 15675636

[46] Yesavage JA, Sheikh JI. 9/Geriatric Depression Scale (GDS). *Clin Gerontol*. 1986; 5(1–2): 165–73. doi: 10.1300/J018v05n01_09

[47] Anderson RM, Funnell MM, Fitzgerald JT, Marrero DG. The Diabetes Empowerment Scale: a measure of psychosocial self-efficacy. *Diabetes Care*. 2000; 23(6): 739–43. doi: 10.2337/diacare.23.6.739 10840988

[48] Garcia AA, Villagomez ET, Brown SA, Kouzekanani K, Hanis CL. The Starr County Diabetes Education Study: development of the Spanish-language diabetes knowledge questionnaire [published correction appears in Diabetes Care 2001 May;24(5):972]. *Diabetes Care*. 2001;24(1):16–21. doi: 10.2337/diacare.24.1.16 11194219

[49] Schmitt A, Gahr A, Hermanns N, Kulzer B, Huber J, Haak T. The Diabetes Self-Management Questionnaire (DSMQ): development and evaluation of an instrument to assess diabetes self-care activities associated with glycaemic control. *Health Qual Life Outcomes*. 2013; 11: 138. doi: 10.1186/1477-7525-11-138 23937988 PMC3751743

[50] Lawton MP, Brody EM. Assessment of older people: self-maintaining and instrumental activities of daily living. *Gerontologist*. 1969;9(3):179–186. 5349366

[51] de Souto Barreto P, Greig C, Ferrandez A-M. Detecting and categorizing frailty status in older adults using a self-report screening instrument. *Arch Gerontol Geriatr*. 2012; 54(3): e249–e54. doi: 10.1016/j.archger.2011.08.003 21889807

[52] Wilhelm-Leen ER, Hall YN, K Tamura M, Chertow GM. Frailty and chronic kidney disease: the Third National Health and Nutrition Evaluation Survey. *Am J Med*. 2009;122(7):664–71.e2. doi: 10.1016/j.amjmed.2009.01.026 19559169 PMC4117255

[53] Feldstein AC, Glasgow RE. A practical, robust implementation and sustainability model (PRISM) for integrating research findings into practice. *Jt Comm J Qual Patient Saf*. 2008;34(4):228–243. doi: 10.1016/s1553-7250(08)34030-6 18468362

[54] Glasgow RE, Battaglia C, McCreight M, Ayele RA, Rabin BA. Making Implementation Science More Rapid: Use of the RE-AIM Framework for Mid-Course Adaptations Across Five Health Services Research Projects in the Veterans Health Administration. *Front Public Health*. 2020; 8:194. Published 2020 May 27. doi: 10.3389/fpubh.2020.00194 32528921 PMC7266866

[55] Keyserling T, Samuel-Hodge C, Ammerman A, et al. A randomized trial of an intervention to improve self-care behaviors of African-American women with type 2 diabetes: impact on physical activity. *Diabetes Care*, 2002; 25(9): 1576–83. doi: 10.2337/diacare.25.9.1576 12196430

[56] Heisler M, Piette JD. "I help you, and you help me": facilitated telephone peer support among patients with diabetes. *Diabetes Educ*. 2005;31(6):869–879. doi: 10.1177/0145721705283247 16288094

[57] Sazlina S, Browning C, Yasin S. Effectiveness of Personalized Feedback Alone or Combined with Peer Support to Improve Physical Activity in Sedentary Older Malays with Type 2 Diabetes: A Randomized Controlled Trial. *Front Public Health*. 2015; 3: 178. doi: 10.3389/fpubh.2015.00178 26217658 PMC4500101

[58] Knox L, Huff J, Graham D, et al. What peer mentoring adds to already good patient care: Implementing the Carpeta Roja peer mentoring program in a well-resourced health care system. *Ann Fam Med*. 2015; 13(Suppl 1): S59–565. doi: 10.1370/afm.1804 26304973 PMC4648130

[59] Woodbury M, Botros M, Kuhnke J, Greene J. Evaluation of a peer-led self-management education programme PEP talk: Diabetes, healthy feet and you. *Int Wound J*. 2013; 10(6): 703–11. doi: 10.1111/iwj.12188 26074389 PMC7950439

[60] Philis-Tsimikas A, Fortmann A, Lleva-Ocana L, Walker C, Gallo L. Peerled diabetes education programs in high-risk Mexican Americans improve glycemic control compared with standard approaches: a Project Dulce promotora randomized trial. *Diabetes Care*. 2011; 34: 1926–31. doi: 10.2337/dc10-2081 21775748 PMC3161298

[61] Long J, Jahnle E, Richardson D, Loewenstein G, Volpp K. Peer mentoring and financial incentives to improve glucose control in African American veterans: a randomized trial. *Ann Intern Med*. 2012; 156(6): 416–24. doi: 10.7326/0003-4819-156-6-201203200-00004 22431674 PMC3475415

[62] Heisler M. Building Peer Support Programs to Manage Chronic Disease: Seven Models for Success. Oakland, CA; 2006.

[63] Ghorob A, Vivas MM, De Vore D, et al. The effectiveness of peer health coaching in improving glycemic control among low-income patients with diabetes: protocol for a randomized controlled trial. *BMC Public Health*. 2011; 11:208. Published 2011 Apr 1. doi: 10.1186/1471-2458-11-208 21457567 PMC3082244

