# Supplementary figures and images for: Older adults using social support to improve self-care (OASIS): Adaptation, implementation and feasibility of peer support for older adults with T2D in appalachia: A feasibility study protocol
Source: PLoS One. 2024 Mar 18;19(3):e0300196. doi: 10.1371/journal.pone.0300196 (PMC10947915; doi:10.1371/journal.pone.0300196)

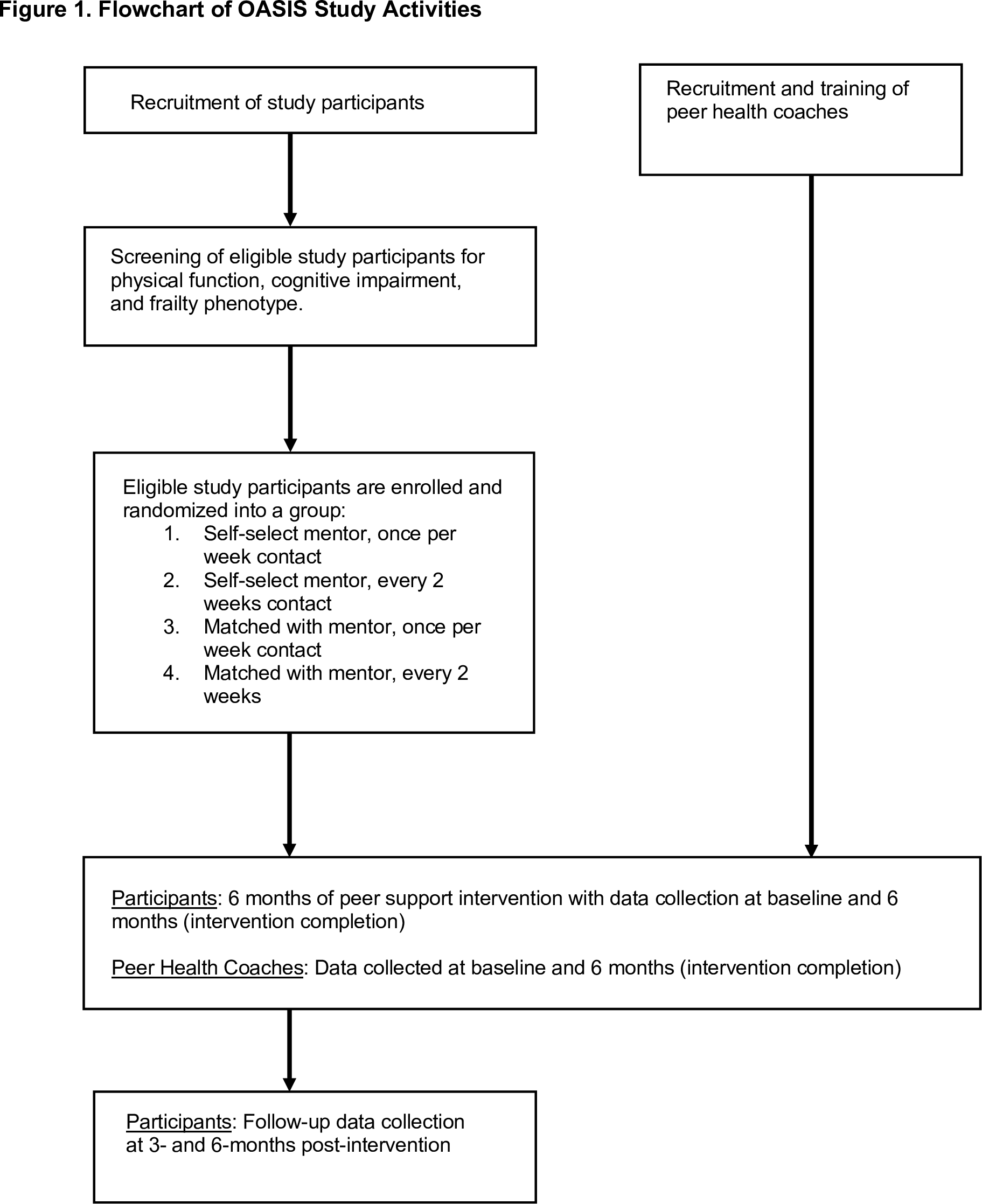

Supplement: S1 Fig — (TIF) [file pone.0300196.s001.tif]
