# Supplementary material for: Older adults using social support to improve self-care (OASIS): Adaptation, implementation and feasibility of peer support for older adults with T2D in appalachia: A feasibility study protocol
Source: PLoS One. 2024 Mar 18;19(3):e0300196. doi: 10.1371/journal.pone.0300196 (PMC10947915; doi:10.1371/journal.pone.0300196)
Supplement: S2 File — (PDF) [file pone.0300196.s003.pdf]

IRB Approval
1/8/2024
IRB # 83904
IRB3

PROTOCOL TYPE (VERSION 4)

0 u
co

Which IRB

(selected) Medical ( ) NonMedical

Protocol Process Type

( ) Exemption
(selected) Expedited (Must be risk level 1)
( ) Full

IMPORTANT NOTE: You will not be able to change your selections for "Which IRB" and "Protocol Process Type" after saving this section. If you select the wrong IRB or Protocol Process Type, you may need to create a new application.

See below for guidance on these options, or refer to ORI's "Getting Started" page. Please contact the Office of Research Integrity (ORI) at 859-257-9428 with any questions prior to saving your selections.

Which IRB

The **Medical IRB** reviews research from the Colleges of:

- Dentistry
- Health Sciences
- Medicine
- Nursing
- Pharmacy and Health Sciences
- and Public Health.

The **Nonmedical IRB** reviews research from the Colleges of:

- Agriculture
- Arts and Sciences
- Business and Economics
- Communication and Information
- Design; Education
- Fine Arts
- Law
- and Social Work

Note: Studies that involve administration of drugs, testing safety or effectiveness of medical devices, or invasive medical procedures must be reviewed by the **Medical IRB** regardless of the college from which the application originates.

Which Protocol Process Type

Under federal regulations, the IRB can process an application to conduct research involving human subjects in one of three ways:

- by exemption certification
- by expedited review.
- by full review;

The investigator makes the preliminary determination of the type of review for which a study is eligible. Please refer to ORI's "Getting Started" page for more information about which activities are eligible for each type of review.

The revised Common Rule expanded exemption certification category 4 for certain secondary research with identifiable information or biospecimens. The regulations no longer require the information or biospecimens to be existing. For more information see the Exemption Categories Tool.

EXPEDITED CERTIFICATION

0 unresolved comment(s)

To Be Completed Only If Protocol is to Receive Expedited Review

Applicability

A. Research activities that (1) present no more than *minimal risk to human subjects, and (2) involve only procedures listed in one or more of the following categories, may be reviewed by the IRB through the expedited review procedure authorized by 45 CFR 46.110 and 21 CFR 56.110. The activities listed should not be deemed to be of minimal risk simply because they are included on this list. Inclusion on this list merely means that the activity is eligible for review through the expedited review procedure when the specific circumstances of the proposed research involve no more than minimal risk to human subjects.
B. The categories in this list apply regardless of the age of subjects, except as noted.
C. The expedited review procedure may not be used where identification of the subjects and/or their responses would reasonably place them at risk of criminal or civil liability or be damaging to the subjects' financial standing, employability, insurability, reputation, or be stigmatizing, unless reasonable and appropriate protections will be implemented so that risks related to invasion of privacy and breach of confidentiality are no greater than minimal.
D. The expedited review procedure may not be used for classified research involving human subjects.
E. IRBs are reminded that the standard requirements for informed consent (or its waiver, alteration, or exception) apply regardless of the type of review—expedited or convened—utilized by the IRB.

**"Minimal risk" means that the probability and magnitude of harm or discomfort anticipated in the research are not greater in and of themselves from those ordinarily encountered in daily life or during the performance of routine physical or psychological examination or tests. 45 CFR 46.102(i)*

Check the appropriate categories that apply to your research project:

☐ Study was originally approved by the full IRB at a convened meeting.

☐ 1) Clinical studies of drugs and medical devices only when condition (a) or (b) is met.

A. Research on drugs for which an investigational new drug application is not required. (Note: Research on marketed drugs that significantly increases the risks or decreases the acceptability of the risks associated with the use of the product is not eligible for expedited review.)
B. Research on medical devices for which (i) an investigational device exemption application is not required*; or (ii) the medical device is cleared/approved for marketing and the medical device is being used in accordance with its cleared/approved labeling.**

* Study must meet one of the IDE Exempt categories listed on the Device Form Attachment.
** An approved Device used in research according to its approved labeling is considered Exempt from IDE requirements.
NOTE: Select Category 1 for compassionate use medical device applications or individual patient expanded access investigational drug applications for which FDA has waived the requirement for full review.

☑ 2) Collection of blood samples by finger stick, heel stick, ear stick, or venipuncture as follows:

A. From healthy, nonpregnant adults who weigh at least 110 pounds. For these subjects, the amounts drawn may not exceed 550 ml in an 8 week period and collection may not occur more frequently than 2 times per week; or
B. From other adults and children* considering the age, weight, and health of the subjects, the collection procedure, the amount of blood to be collected, and the frequency with which it will be collected. For these subjects, the amount drawn may not exceed the lesser of 50 ml or 3 ml per kg in an 8 week period and collection may not occur more frequently than 2 times per week.

NOTE: Intravenous (IV), Port, Central, or any other lines are NOT eligible under this category even if the research involves "minimal risk".

*In Kentucky, "child/children" refers to all individuals less than 18 years of age unless the individual(s) is/are legally emancipated. (See Informed Consent SOP for discussion of "Emancipated Individuals" under Kentucky state law.) Individuals less than 18 years of age who are not emancipated meet the federal definition for "child" (e.g., DHHS, FDA, and U.S. Department of Education). Children are defined in the HHS regulations as "persons who have not attained the legal age for consent to treatments or procedures involved in the research, under the applicable law of the jurisdiction in which the research will be conducted." If conducting research outside the state of Kentucky, you are responsible for complying with applicable state law.

☐ 3) Prospective collection of biological specimens for research purposes by noninvasive means. Examples:

A. Hair and nail clippings in a nondisfiguring manner;
B. Deciduous teeth at time of exfoliation or if routine patient care indicates a need for extraction;
C. Permanent teeth if routine patient care indicates a need for extraction;
D. Excreta and external secretions (including sweat);
E. Uncannulated saliva collected either in an unstimulated fashion or stimulated by chewing gumbase or wax or by applying a dilute citric solution to the tongue;
F. placenta removed at delivery;
G. Amniotic fluid obtained at the time of rupture of the membrane prior to or during labor;
H. Supra- and subgingival dental plaque and calculus, provided the collection procedure is not more invasive than routine prophylactic scaling of the teeth and the process is accomplished in accordance with accepted prophylactic techniques;
I. Mucosal and skin cells collected by buccal scraping or swab, skin swab, or mouth washings;
J. Sputum collected after saline mist nebulization.

☐ 4) Collection of data through noninvasive procedures (not involving general anesthesia or sedation) routinely employed in clinical practice, excluding procedures involving x-rays or microwaves. Where medical devices are employed, they must be cleared/approved for marketing. (Studies intended to evaluate the safety and effectiveness of the medical device are not generally eligible for expedited review, including studies of cleared medical devices for new indications.) Examples:

A. Physical sensors that are applied either to the surface of the body or at a distance and do not involve input of significant amounts of energy into the subject or an invasion of the subject's privacy;
B. Weighing or testing sensory acuity;
C. Magnetic resonance imaging;
D. electrocardiography, electroencephalography, thermography, detection of naturally occurring radioactivity, electroretinography, ultrasound, diagnostic infrared imaging, doppler blood flow, and echocardiography;
E. moderate exercise, muscular strength testing, body composition assessment, and flexibility testing where appropriate given the age, weight, and health of the individual.

☐ 5) Research involving materials (data, documents, records, or specimens) that have been or will be collected solely for non-research purposes (such as medical treatment or diagnosis) as well as research involving existing information or specimens that were previously collected for research purposes, provided they were not collected for the currently proposed research. (Note: Some research in this category may qualify for Exempt review. This listing refers only to research that is not exempt.)
(Note: If submission includes materials previously collected for either non-research or research purposes in a protocol for which IRB approval expired, you may check Category 5. However, a separate category must also be selected for prospective collection of data/specimens obtained solely for research purposes)

☐ 6) Collection of data from voice, video, digital, or image recordings made for research purposes.

☑ 7) Research on individual or group characteristics or behavior (including, but not limited to, research on perception, cognition, motivation, identity, language, communication, cultural beliefs or practices, and social behavior) or research employing survey, interview, oral history, focus group, program evaluation, human factors evaluation, or quality assurance methodologies. (Note: Some research in this category may be exempt from the HHS regulations for the protection of human subjects. This listing refers only to research that is not exempt.)

Modification Request Section

1 unresolved comment(s)

***** If this modification changes the scope of your activities to include COVID-19 related research, please insert "COVID19" at the start of your Project and Short Titles.*****

Select One:
- (selected) This modification does not increase risk to study participants.
- ( ) This modification may or will increase risk to study participants.

Is this modification request due to an Unanticipated Problem/Adverse Event, or Protocol Violation?
( ) Yes (selected) No

In your professional opinion, does this modification involve information that might relate to a subject's willingness to continue to take part in the research?
( ) Yes (selected) No

If yes, state how the information will be communicated to subjects (i.e., re-consent, send letter, etc.):

For each proposed modification, include a justification.

Example: Jane Doe, MD, is being added as co-investigator because she has expertise with the subjects on this protocol. She has completed human subject protections training, and is authorized to obtain consent.

Addition of post peer coach training written examination. This document was screened for and is in compliance with IRB standard for reading level (Flesch-Kincaid Grade Level 5.4). Second modification was made to the peer coach training document, where a dental health and diabetes flyer was added, and content changes were made to the 'ABCs' of diabetes flyer.

PROJECT INFORMATION

0 unresolved comment(s)

Title of Project: (Use the exact title listed in the grant/contract application, if applicable).
If your research investigates any aspect of COVID-19, please include "COVID19" at the beginning of your Project Title and Short Title

Older Adults using Social Support to Improve Self-Care (OASIS): Adaptation, Implementation, and Feasibility of Peer Support for Older Adults with T2DM in Appalachia.

Short Title Description
Please use a few key words to easily identify your study - this text will be displayed in the Dashboard listing for your study.

OASIS: Peer Support for T2DM in Appalachia

Anticipated Ending Date of Research Project: 1/31/2025

Maximum number of human subjects (or records/specimens to be reviewed) 108

After approval, will the study be open to enrollment of new subjects or new data/specimen collection? (•) Yes ( ) No

Are you requesting that the UK IRB serve as the lead IRB for a multi-site study, or that the UK IRB defer review to another IRB? [Click here for "IRB Reliance" help]
( ) Yes (•) No
If "Yes," before completing your IRB application, fill out the Reliance Request Form and submit it to irbreliance@uky.edu.

PI CONTACT INFORMATION

0 unresolved comment(s)

Principal Investigator (PI) role for E-IRB access
The PI is the individual holding primary responsibility on the research project with the following permissions on the E-IRB application:

1. Read;
2. write/edit;
3. receive communications; and
4. submit to the IRB (IR, CR, MR, Other Review*).

If research is being submitted to or supported by an extramural funding agency such as NIH, a private foundation or a pharmaceutical/manufacturing company, the PI listed on the grant application or the drug protocol must be listed as PI here.

Please fill in any blank fields with the appropriate contact information (gray shaded fields are not editable). Required fields left blank will be highlighted in pink after you click "Save".

To change home and work addresses, go to myUK and update using the Employee Self Service (ESS) portal. If name has changed, the individual with the name change will need to submit a 'Name Change Form' to the Human Resources Benefits Office for entering into SAP. The new name will need to be associated with the individual's Link Blue ID in SAP before the change is reflected in E-IRB. Contact the HR Benefits Office for additional information.

The Principal Investigator's (PI) contact information is filled in automatically based on who logged in to create the application.

If you are not the Principal Investigator, do NOT add yourself as study personnel.
To change the PI contact information on an application in Researcher edit status:

- click "Change Principal Investigator";
- search for the PI's name using the search feature;
- click "Select" by the name of the Principal Investigator, then "Save Contact Information".

You will automatically be added as study personnel with editing permissions to continue editing the application.

Change Principal Investigator:

First Name: Brittany
Last Name: Smalls
Middle Name: L
Department: Family and Community Medici…
PI's Employee/Student ID#: 12224507
PI's Telephone #: 859-323-4916
PI's e-mail address: Brittany.Smalls@uky.edu
PI is R.N. ○ Yes ● No

Room# & Bldg: 2195 Harrodsburg Rd Ste125
Speed Sort#: 40504
Dept Code: 7H460
Rank: Associate Professor
Degree: PhD
PI's FAX Number:
HSP Trained: Yes
HSP Trained Date: 1/1/2023
RCR Trained: Yes

Do you, the PI, have a significant financial interest related to your responsibilities at the University of Kentucky (that requires disclosure per the UK administrative regulation 7:2)?

○ Yes ● No

RISK LEVEL

0 unresolved comment(s)

Indicate which of the categories listed below accurately describes this protocol

- ◉ (Risk Level 1) Not greater than minimal risk
- ○ (Risk Level 2) Greater than minimal risk, but presenting the prospect of direct benefit to individual subjects
- ○ (Risk Level 3) Greater than minimal risk, no prospect of direct benefit to individual subjects, but likely to yield generalizable knowledge about the subject's disorder or condition.
- ○ (Risk Level 4) Research not otherwise approvable which presents an opportunity to understand, prevent, or alleviate a serious problem affecting the health or welfare of subjects.

*"Minimal risk" means that the probability and magnitude of harm or discomfort anticipated in the research are not greater in and of themselves from those ordinarily encountered in daily life or during the performance of routine physical or psychological examination or tests.

*****For Expedited and Exempt Applications, the research activities must be Risk Level 1 (no more than minimal risk to human subjects).*****

Refer to UK's guidance document on assessing the research risk for additional information.

SUBJECT DEMOGRAPHICS **0 unresolved comment(s)**

Age level of human subjects: (i.e., 6 mths.; 2yrs., etc..) 18 to 99

Study Population:

Describe the characteristics of the subject population, including age range, gender, ethnic background and health status. Identify the criteria for inclusion and exclusion.

Provide the following information:

- A description of the subject selection criteria and rationale for selection in terms of the scientific objectives and proposed study design;
- A compelling rationale for proposed exclusion of any sex/gender or racial/ethnic group;
- Justification for the inclusion of vulnerable groups such as children, prisoners, adults with impaired consent capacity, or others who may be vulnerable to coercion or undue influence.

Please consider these resources:
NIH Diversity Policy
FDA Diversity Guidance

Inclusion criteria for peer participants (n=76)
1. Confirmed diagnosis of T2D via point-of-care HbA1C assessment.
2. Residence in Appalachian KY
3. HbA1c >= 7.5% in the previous 6 months
4. Age >= 55 years

Inclusion Criteria for peer coaches (n=25)
1. Confirmed diagnosis of T2D via point-of-care HbA1C assessment.
2. Residence in Appalachian KY
3. HbA1c<7.5% at time of enrollment
4. Age >= 55 years
5. Diagnosed >=2 years

Inclusion criteria for stakeholders (n=5-7)
Age>=18
This is a purposive sample of stakeholders (community leaders, older adults with T2D, representatives from BRADD organizations [Aging on Aging case worker, Community Action], who will provide feedback, ensure fidelity, and acceptability of the final evidence based intervention. Stakeholders will not have HbA1C checked. These stakeholders will participate in interview at study startup, mid intervention and at the conclusion of the intervention. A sample of randomly selected peer health coaches from each of the four conditions will be included in the final team meeting following the end of the intervention. Also, peer participants from each condition will complete the same survey and complete exit interviews to explore perceived and actual factors underscoring participant satisfaction with the EBI and its components.
This study will not involve medical records.

Attachments

Indicate the targeted/planned enrollment of the following members of minority groups and their subpopulations. Possible demographic sources: Census Regional Analyst Edition, Kentucky Race/Ethnic Table, Kentucky Population Data.
(Please note: The IRB will expect this information to be reported at Continuation Review time for Pre-2019 FDA-regulated Expedited review and Full review applications):

Participant Demographics

	Cisgender Man	Cisgender Woman	TGNB/TGE	Unknown/Not Reported
American Indian/Alaskan Native:	1	1		
Asian:	0	2		
Black/African American:	4	9		
Latinx:	3	5		
Native Hawaiian/Pacific Islander:				
White:	40	43		
American Arab/Middle Eastern/North African:				
Indigenous People Around the World:				
More than One				

Race:				
Unknown or Not Reported:				

If unknown, please explain why:

Above numbers are estimates based on Kentucky Census data

Indicate the categories of subjects and controls to be included in the study. You may be required to complete additional forms depending on the subject categories which apply to your research. If the study does not involve direct intervention or direct interaction with subjects, (e.g., record-review research, outcomes registries), do not check populations which the research does not specifically target. For example: a large record review of a diverse population may incidentally include a prisoner or an international citizen, but you should not check those categories if the focus of the study has nothing to do with that status.

Check All That Apply (at least one item must be selected)

- ☐ Children (individuals under age 18)
- ☐ Wards of the State (Children)
- ☐ Emancipated Minors
- ☐ Students
- ☐ College of Medicine Students
- ☐ UK Medical Center Residents or House Officers
- ☐ Impaired Consent Capacity Adults
- ☐ Pregnant Women/Neonates/Fetal Material
- ☐ Prisoners
- ☐ Non-English Speaking (translated long or short form)
- ☐ International Citizens
- ☑ Normal Volunteers
- ☐ Military Personnel and/or DoD Civilian Employees
- ☐ Patients
- ☑ Appalachian Population

ADDITIONAL INFORMATION:

Please visit the IRB Survival Handbook for more information on:

- Children/Emancipated Minors
- Students as Subjects
- Prisoners
- Impaired Consent Capacity Adults
- Economically or Educationally Disadvantaged Persons

Other Resources:

- UKMC Residents or House Officers [see requirement of GME]
- Non-English Speaking [see also the E-IRB Research Description section on this same topic]
- International Citizens [DoD SOP may apply]
- Military Personnel and/or DoD Civilian Employees

Assessment of the potential recruitment of subjects with impaired consent capacity (or likelihood):

☐ Check this box if your study does NOT involve direct intervention or direct interaction with subjects (e.g., record-review research, secondary data analysis). If there is no direct intervention/interaction you will not need to answer the impaired consent capacity questions.

Does this study focus on adult subjects with any conditions that present a high *likelihood* of impaired consent capacity or *fluctuations* in consent capacity? (see examples below)

○ Yes ◉ No

If Yes and you are not filing for exemption certification, go to "Form T", complete the form, and attach it using the button below.

Examples of such conditions include:

- Traumatic brain injury or acquired brain injury
- Severe depressive disorders or Bipolar disorders
- Schizophrenia or other mental disorders that
  involve serious cognitive disturbances
- Stroke
- Developmental disabilities
- Degenerative dementias
- CNS cancers and other cancers with possible CNS involvement
- Late stage Parkinson's Disease
- Late stage persistent substance dependence
- Ischemic heart disease
- HIV/AIDS
- COPD
- Renal insufficiency
- Diabetes
- Autoimmune or inflammatory disorders
- Chronic non-malignant pain disorders
- Drug effects
- Other acute medical crises

Attachments

INFORMED CONSENT/ASSENT PROCESS/WAIVER

0 unresolved comment(s)

For creating your informed consent attachment(s), please download the most up-to-date version listed in "All Templates" under the APPLICATION LINKS menu on the left, and edit to match your research project.

Additional Resources:

- Informed Consent/Assent Website
- Waiver of Consent vs. Waiver of Signatures
- Sample Repository/Registry/Bank Consent Template

Consent/Assent Tips:

- If you have multiple consent documents, be sure to upload each individually (not all in a combined file).
- If another site is serving as the IRB for the project, attach the form as a “Reliance Consent Form” so the document will not receive a UK IRB approval stamp; the reviewing IRB will need to stamp the consent forms.
- Changes to consent documents (e.g., informed consent form, assent form, cover letter, etc...) should be reflected in a 'tracked changes' version and uploaded separately with the Document Type "Highlighted Changes".
- It is very important that only the documents you wish to have approved by the IRB are attached; DELETE OUTDATED FILES -- previously *approved* versions will still be available in Protocol History.
- Attachments that are assigned a Document Type to which an IRB approval stamp applies will be considered the version(s) to be used for enrolling subjects once IRB approval has been issued.
  Document Types that do NOT get an IRB approval stamp are:
  - "Highlighted Changes",
  - "Phone Script", and
  - "Reliance Consent Form",
  - "Sponsor's Sample Consent Form".

How to Get the Section Check Mark

1. You must:

   a) provide a response in the text box below describing how investigators will obtain consent/assent, and
   b) check the box for at least one of the consent items and/or check mark one of the waivers

2. If applicable attach each corresponding document(s) **as a read-only PDF**.
3. If you no longer need a consent document approved (e.g., closed to enrollment), or, the consent document submitted does not need a stamp for enrolling subjects (e.g., umbrella study, or sub-study), only select "Stamped Consent Doc(s) Not Needed".
4. After making your selection(s) be sure to scroll to the bottom of this section and SAVE your work!

Check All That Apply

- [x] Informed Consent Form (and/or Parental Permission Form and/or translated short form)
- [ ] Assent Form
- [ ] Cover Letter (for survey/questionnaire research)
- [ ] Phone Script
- [ ] Informed Consent/HIPAA Combined Form
- [ ] Debriefing and/or Permission to Use Data Form
- [ ] Reliance Consent Form
- [ ] Sponsor's sample consent form for Dept. of Health and Human Services (DHHS)-approved protocol
- [ ] Stamped Consent Doc(s) Not Needed

Attachments

Attach Type	File Name
Informed Consent/Parental Permission	ElectronicConsentStakeholders_ (3).pdf
Informed Consent/Parental Permission	OASIS_PC_Consent.pdf
Informed Consent/Parental Permission	ElectronicConsentPeerCoaches_Clean.pdf
Informed Consent/Parental Permission	OASIS_Stakeholder_Consent.pdf
Informed Consent/Parental Permission	OASIS_PeerP_ElectronicConsent_CLEAN.pdf

Informed Consent Process:

Using active voice, describe how investigators will obtain consent/assent. Include:

- the circumstances under which consent will be sought and obtained
- the timing of the consent process (including any waiting period between providing information and obtaining consent)
- who will seek consent
- how you will minimize the possibility of coercion or undue influence
- the method used for documenting consent
- if applicable, who is authorized to provide permission or consent on behalf of the subject
- if applicable, specific instruments or techniques to assess and confirm potential subjects' understanding of the information

Note: all individuals authorized to obtain informed consent should be designated as such in the E-IRB "Study Personnel" section of this application.

Special considerations may include:

- Obtaining consent/assent for special populations such as children, prisoners, or people with impaired decisional capacity
- *Research Involving Emancipated Individuals*
  If you plan to enroll some or all prospective subjects as emancipated, consult with UK legal counsel **prior to submitting this application to the IRB**. Include research legal counsel's recommendations in the "Additional Information" section as a separate document.
- *Research Involving Non-English Speaking Subjects*
  For information on inclusion of non-English speaking subjects, or subjects from a foreign culture, see IRB Application Instructions for Recruiting Non-English Speaking Participants or Participants from a Foreign Culture.
- *Research Repositories*
  If the purpose of this submission is to establish a research repository describe the informed consent process. For guidance regarding consent issues, process approaches, and sample language see the Sample Repository/Registry/Bank Consent Template.

All study personnel will be CITI/HSP certified. Individuals must provide consent prior to participating in the intervention. Individuals will be consented in-person prior to the start of the intervention, and will be able to participate immediately after giving consent. Study personnel will read the informed consent to participants if they are unable to read, and will check for comprehension by asking knowledge check questions such as "Do you understand what you will be asked to do?" "After learning about the study, do you still want to participate?" Obtaining e-consent via REDCap will be an option for participants if needed.

Should any subjects have complaints, they will be encouraged to contact the Principal Investigator, Brittany Smalls, PhD at (859) 323-4916 or brittany.smalls@uky.edu. They can also send a letter to Dr. Smalls at 2195 Harrodsburg Road, Suite 125, Lexington, KY 40504. They will also be provided with the contact information for University of Kentucky's ORI.

☐ Request for Waiver of Informed Consent Process

If you are requesting IRB approval to waive the requirement for the informed consent process, or to alter some or all of the elements of informed consent, complete, Section 1 and Section 2 below.

Note: The IRB does not approve waiver or alteration of the consent process for greater than minimal risk research, except for planned emergency/acute care research as provided under FDA regulations. Contact ORI for regulations that apply to single emergency use waiver or acute care research waiver (859-257-9428).

SECTION 1.

Check the appropriate item:

☐ I am requesting a waiver of the requirement for the informed consent process.

☐ I am requesting an alteration of the informed consent process.
If you checked the box for this item, describe which elements of consent will be altered and/or omitted, and justify the alteration.

SECTION 2.

Explain how each condition applies to your research.

a) The research involves no more than minimal risk to the subject.

b) The rights and welfare of subjects will not be adversely affected.

c) The research could not practicably be carried out without the requested waiver or alteration.

d) Whenever possible, the subjects or legally authorized representatives will be provided with additional pertinent information after they have participated in the study.

e) If the research involves using or accessing identifiable private information or identifiable biospecimens, the research could not practicably be carried out without using such information or biospecimens in an identifiable format.

- Private information/specimens are “identifiable” if the investigator may ascertain the identity of the subject or if identifiers are associated with the information (e.g., medical records). This could be any of the 18 HIPAA identifiers including dates of service.
- If not using identifiable private information or identifiable biospecimens, insert N/A below.

☐ Request for Waiver of Signatures

If you are requesting IRB approval to waive the requirement for signatures on informed consent forms, **your research activities must fit into one of three regulatory options**:

1. The only record linking the participant and the research would be the consent document, and the principal risk would be potential harm resulting from a breach of confidentiality (e.g., a study that involves participants who use illegal drugs).
2. The research presents no more than minimal risk to the participant and involves no procedures for which written consent is normally required outside of the research context (e.g., a cover letter on a survey, or a phone script).
3. The participant (or legally authorized representative) is a member of a distinct cultural group or community in which signing forms is not the norm, the research presents no more than minimal risk to the subject, and there is an appropriate alternative mechanism for documenting that informed consent was obtained.

Select the option below that best fits your study.

*If the IRB approves a waiver of signatures, participants must still be provided oral or written information about the study. To ensure you include required elements in your consent document, use the **Cover Letter Template** as a guide. There is an English and a Spanish version.*

○ **Option 1**

Describe how your study meets these criteria:

a) The only record linking the participant and the research would be the consent document:

b) The principal risk would be potential harm resulting from a breach of confidentiality (i.e., a study that involves subjects who use illegal drugs).

Under this option, each participant (or legally authorized representative) must be asked whether (s)he wants to sign a consent document; if the participant agrees to sign a consent document, only an IRB approved version should be used.

○ **Option 2**

Describe how your study meets these criteria:

a) The research presents no more than minimal risk to the participant:

b) Involves no procedures for which written consent is normally required outside of the research context (i.e. a cover letter on a survey, or a phone script):

○ **Option 3**

Describe how your study meets these criteria:

a) The subject (or legally authorized representative) is a member of a distinct cultural group or community in which signing forms is not the norm.

b) The research presents no more than minimal risk to the subject.

c) There is an appropriate alternative mechanism for documenting that informed consent was obtained.

STUDY PERSONNEL

0 unresolved comment(s)

Do you have study personnel who will be assisting with the research?
After selecting 'Yes' or 'No' you must click the 'Save Study Personnel Information' button.

◉ Yes ○ No

Manage Study Personnel

Identify other study personnel assisting in research project:

- The individual listed as PI in the 'PI Contact Information' section should NOT be added to this section.
- If the research is required for a University of Kentucky academic program, the faculty advisor is also considered study personnel and should be listed below. ***Residents and students who are PI's are encouraged to designate the faculty advisor or at least one other individual as a contact with an editor role (DP).***
- Role: DP = Editor (individual can view, navigate, and edit the application for any review phase (IR, CR/FR, MR) or 'Other Review", and submit Other Reviews on behalf of the PI.)
- Role: SP = Reader (individual can view and navigate through the currently approved application only.)

To add an individual via the below feature:

- Search for personnel;
- Click "select" by the listing for the person you want to add;
- For each person, specify responsibility in the project, whether authorized to obtain informed consent, AND denote who should receive E-IRB notifications (contact status).

NOTE: Study personnel must complete human subject protection (HSP) and Responsible Conduct of Research (RCR) training before implementing any research procedures. For information about training requirements for study personnel, visit UK's HSP FAQ page, the RCR Getting Started page, or contact ORI at 859-257-9428. If you have documentation of current HSP training other than that acquired through UK CITI, you may submit it to ORI (HSPTrainingSupport@uky.edu) for credit.

Study personnel assisting in research project:

Last Name	First Name	Responsibility In Project	Role	A C	Contact	Degree	StatusFlag	(HSP)	(HSP)Date	(RCR)	Removed?	Last Updated	SFI	Active
Douthitt	Key	Medical Supervisor	SP	N	N	MD	P	Y	11/01/2023	Y	N	11/06/2023	N	Y
Gonzabato	Nelson	Data Analysis/Processing	SP	N	N		P	Y	08/25/2023	Y	N	08/29/2023	N	Y
Kruse-Diehr	Aaron	Co-Investigator	DP	Y	Y	PhD	P	Y	02/21/2022	Y	N	12/08/2022	N	Y
McLouth	Christopher	Data Analysis/Processing	SP	N	N	PhD	P	Y	07/25/2022	Y	N	11/09/2022	N	Y
Ortz	Courtney	Project Assistance/Support	DP	N	Y		P	Y	01/30/2022	Y	N	11/09/2022	N	Y
Taylor	Zoe	Study Coordinator	DP	Y	Y		P	Y	05/12/2022	Y	N	06/05/2023	N	Y

RESEARCH DESCRIPTION

0 unresolved comment(s)

You may attach a sponsor's protocol pages in the "Additional Information" section and refer to them where necessary in the Research Description. However, each prompt that applies to your study should contain at least a summary paragraph.

Pro Tips:

- **Save your work often to avoid losing data.**
- **Use one of the attachment buttons in this section or under the Additional Information section to include supplemental information with your application. During the document upload process, you will be able to provide a brief description of the attachment.**

Background

Include a brief review of existing literature in the area of your research. You should identify gaps in knowledge that should be addressed and explain how your research will address those gaps or contribute to existing knowledge in this area. For interventional research, search PubMed and ClinicalTrials.gov for duplicative ongoing and completed trials with same condition and intervention(s).

According to the Administration for Community Living, 16% (54.1 million) of the United States population is comprised of people aged greater than or equal to 65 years, a percentage poised to rise to 21.6% (80.8 million) by 2040. In Kentucky, older adults account for an estimated 19% of the population, which reflects a 32% increase between 2009 and 2019. The rising number of older adults coincides with increased prevalence of chronic illness, including Type 2 diabetes (T2D). At present, an estimated 29% of older adults (greater than or equal to 65 years) have diagnosed or undiagnosed T2D. Moreover, older adults living with T2D also have two times the annual healthcare expenditures as their younger counterparts— $13,239 versus $6,675, respectively.

Appropriate self-care behaviors are necessary for optimal clinical outcomes in T2D, accounting for 90% of variance in glycemic control. Older adults living with T2D have more difficulty adhering to self-care regimens, and social environment factors account for up to 85% of their self-care nonadherence compared to younger adults living with T2D. Notable social environment factors include self-efficacy, distress, and lack of social support. Specifically, social support plays a significant role in older adults' T2D self-management.

Social support is an important social environment factor to target in people with T2D due to its well-established relationship with self-care adherence. Older rural-dwelling adults diagnosed with chronic disease have worse social function and emotional well-being than older adults living in urban areas. Although social support has been shown to have a significant, independent relationship with T2DM self-care, the extent and type of social support that may be available in rural communities might either positively or negatively impact older rural-dwelling adults' self-care. The type and quality of social support can either reinforce or mitigate structural and psychosocial health determinants—such as healthcare mistrust, cultural health beliefs, and access to healthcare—that can influence self-care behaviors and beliefs. Nevertheless, there remains a body of literature that substantiates the influence of social support on T2DM self-care as well as the disproportionate burden of T2D in older adults.

In addition, frequency of desired social contact is also important for older adults. Previous research shows that more frequent social contact can mitigate depressive symptoms, frailty, and cognitive decline. Frequencies of contact ranging from daily or weekly to monthly are each associated with health and wellbeing in older adults. When designing and administering social support interventions in older adults, ensuring the appropriate type of support and the necessary frequency of contact are both important considerations to providing appropriate social support for T2D self-management.

This study focuses on peer support. Peer support provides a mechanism for creating a social network that augments existing social supports. Rural Appalachia Kentucky is characterized by close knit communities and lends itself to the use of structured social support interventions, such as peer support, to promote desired health behaviors.

Objectives

List your research objectives. Please include a summary of intended research objectives in the box below.

All stages of the proposed study are guided by the ADAPT-ITT model, an adaptations framework used in implementation science research to inform evidence based intervention (EBI) selection and adaptation for cultural and contextual relevance in new populations.

The objectives of this study are twofold. One is to evaluate the pragmatic implementability of a peer support intervention, peer health coaching, and the other is to use a 2x2 factorial design to test specific components of a peer support intervention—peer health coaching—to determine which components are most effective at promoting self-care behaviors and improving glycemic control among older adults living in Appalachian Kentucky. These two parts will occur concurrently.

To better understand implementability of the EBI, as well as each of the four strategies tested in the 2 x 2 factorial design, we will assess contextual factors that influence EBI adoption as well as implementation outcomes using the Practical, Robust Implementation and Sustainability Model (PRISM), a framework that incorporates RE-AIM dimensions (i.e., reach, effectiveness, adoption, implementation, and maintenance). We will use a novel iterative approach to RE-AIM, wherein team members (stakeholders) will collectively refine proposed operationalizations of RE-AIM dimensions and then, at project mid-point, identify RE-AIM dimensions

needing added attention and set goals and strategies to improve progress on them, including potentially adapting or rejecting and replacing indicators to better capture necessary outcomes data.

Study Design

Describe and explain the study design (e.g., observational, secondary analysis, single/double blind, parallel, crossover, deception, etc.).

- *Clinical Research*: Indicate whether subjects will be randomized and whether subjects will receive any placebo.
- *Community-Based Participatory Research*: If you are conducting community-based participatory research (CBPR), describe strategies for involvement of community members in the design and implementation of the study, and dissemination of results from the study.
- *Qualitative research*: Indicate ranges where flexibility is needed, if a fixed interview transcript is not available, describe interview topics including the most sensitive potential questions.
- *Research Repositories*: If the purpose of this submission is to establish a Research Repository (bank, registry) and the material you plan to collect is already available from a commercial supplier, clinical lab, or established IRB approved research repository, provide scientific justification for establishing an additional repository collecting duplicate material. Describe the repository design and operating procedures. For relevant information to include, see the UK Research Biospecimen Bank Guidance or the UK Research Registry Guidance.

There are two parts to this study that are happening concurrently. The first part is the interviews with stakeholders. The second part is the intervention with peer coaches and peer participants.

This research is guided by the ADAPT-ITT model, an adaptations framework used in implementation science research to inform evidence based intervention (EBI) selection and adaptation for cultural and contextual relevance in new populations. Specifically for this study, we will focus on phases 5-8 where we will gather feedback from experts on draft 1 of the adapted EBI, integrate the feedback, check for fidelity and acceptability and draft final EBI, train staff to implement the final EBI and conduct a pilot implementation trial.
Stakeholders:
We will employ a qualitative design using purposive sampling whereby we will conduct brief interviews with a set of diverse program stakeholders (including clinical and community partners, and research staff) with discrete implementation responsibilities. To better understand implementability of the EBI, as well as each of the four strategies tested in the 2 x 2 factorial design, we will assess contextual factors that influence EBI adoption as well as implementation outcomes using the Practical, Robust Implementation and Sustainability Model (PRISM), a framework that incorporates RE-AIM dimensions (i.e., reach, effectiveness, adoption, implementation, and maintenance). We will use a novel iterative approach to RE-AIM, wherein team members (both researchers and implementers) will collectively refine proposed operationalizations of RE-AIM dimensions and then, at project mid-point, identify RE-AIM dimensions needing added attention and set goals and strategies to improve progress on them, including potentially adapting or rejecting and replacing indicators to better capture necessary outcomes data. This iterative use of RE-AIM will ensure that any midcourse corrections are informed by pragmatic need and are documented accordingly.

Interviews will be conducted at three timepoints: (1) at beginning of implementation, (2) at implementation midpoint, and (3) upon conclusion. We will collect both qualitative and quantitative data to evaluate RE-AIM dimensions upon project conclusion. Utilizing a multiple timepoint data collection design will promote both data triangulation and richness of data to better understand both planned and unplanned facilitators, barriers, and adaptations throughout the implementation process.

Peer coaches and peer participants
The EBI used is peer health coaching. This study utilizes a 2x2 factorial design. The independent variables for the factorial design are (1) how the peer coach is selected (self-selected by participant vs matched) and (2) frequency of contact (once per week vs every 2 weeks). We will evaluate effectiveness of each group using HbA1c as the primary outcome while also evaluating self-care activities and T2D-related psychosocial factors as secondary outcomes.

The proposed study will provide essential information on how to improve support to a population that is at increasing risk of experiencing morbidity and mortality as a result of T2DM. The findings from this study will be used to test the effectiveness of the refined intervention in a larger, adequately powered study. Data collected on implementation science components, feasibility, and acceptability will be used to scale up the peer health coach intervention. Lastly, the subsequent study will assess cost effectiveness of the large-scale intervention (e.g., economic evaluation of cost, implementation specific cost assessment) as well as considerations for sustainability.

The findings from the proposed study will be disseminated using the following platforms: (1) submission of abstracts to national, regional, and local scientific meetings, (2) publication in peer review journals, and (3) present findings to study participants in a town hall meeting and in printed format.

Attachments

Subject Recruitment Methods & Advertising

Describe how the study team will identify and recruit subjects. Please consider the following items and provide additional information as needed so that the IRB can follow each step of the recruitment process.

- How will the study team identify potential participants?
- Who will first contact the potential subjects, and how?
- Will you use advertisements? If so, how will you distribute those?

- How and where will the research team meet with potential participants?
- If applicable, describe proposed outreach programs for recruiting women, minorities, or disparate populations.
- How you will minimize undue influence in recruitment?
- Attach copies of all recruiting and advertising materials (emails, verbal scripts, flyers, posts, messages, etc.).

For additional information on recruiting and advertising:

- IRB Application Instructions - Advertisements
- PI Guide to Identification and Recruitment of Human Subjects for Research

Recruitment of peer participants.
Participants will be recruited from community-based organizations located in the Barren River Area Development District (BRADD) (letter of support in research sites tab). Eligibility criteria for peer participants include: (1) confirmed diagnosis of T2D via point-of-care HbA1c assessment; (2) residence in Appalachia Kentucky; (3) HbA1c greater than or equal to 7.5% in the previous 6 months; (4) age greater than or equal to 55 years. Study personnel will not access medical records as participants who are referred will have a relationship with the individuals doing the referring such that they know the participants HbA1c level history. All individuals who are determined to be eligible for the study will then be screened for cognitive impairment using the Montreal Cognitive Assessment-Basic (MoCA-B) to ensure that study participants have the capacity to engage in study activities. After passing the screening assessment, participants will be randomized into one of four groups: 1) self-select coach, once per week contact; 2) self-select coach, every 2 weeks contact; 3) matched with peer coach, once per week contact; and 4) matched with peer coach, every 2 weeks contact.

Recruitment, screening, and training of peer health coaches.
Potential peer coaches will be identified by referral of healthcare providers in the BRADD area. Coaches will be required to have T2D, diagnosed for at minimum 2 years, but their HbA1c will be less than 7.5% at time of enrollment. They will also be greater than or equal to 55 years of age as well as a resident of Appalachia Kentucky. We will mail out recruitment letters that will explain the study and provide participants a number to call if they are interested in participating. Study personnel will conduct a pre-screening assessment with potential participants who indicate interest in the study. If eligibility criteria are met, the screening/enrollment visit will be scheduled. As part of the coach screening interview, psychosocial status will be assessed using the psychological scales of the Wallston General Perceived Competence Scale, Campbell Personal Competence Index, Carkhuff Communication and Discrimination Skills Inventories, and the Applied Knowledge Assessment (AKA) scale. The PI will make a determination of competence, maturity, emotional stability, and verbal communication skills after overall assessment during the screening interview and training. Post-training, peer coaches will need to pass an examination that demonstrates their diabetes knowledge and application of proper self-care activities.

Recruitment and enrollment of peer coaches/peer participants will occur in 3 waves. Within each wave, each coach will be assigned all of their peer participants at one time to ensure that intervention activities occur within the same time period. Peers and coaches will attend an introductory session together, during which the coaching process will be discussed, including time commitment, roles, responsibilities, benefits, and ground rules. At that time, peer participants who are randomized to self-select their coaches will have the opportunity to review peer coach profiles and rank their top 3 coaches to be matched. Once coaches and peers have been selected, the quads (coach with 3 peers) will have the opportunity to ask questions and make informed decisions about their ability to fully participate in the intervention. If face-to-face meetings are not possible for all members of the “quad,” phone or a form of video meeting will be attempted.

Recruitment Flyers for both peer participants and coaches have been attached.

Recruitment of Stakeholders for EBI adaptations
Stakeholders will be those who are community leaders, older adults with T2D, representatives from BRADD organizations (Aging on Aging case worker, Community Action). They will not have their HBA1C checked and will only participate in the interviews.

Attachments

Attach Type	File Name
Advertising	OASIS_Flyers-STAMPED.pdf
Advertising	87024_Advertising_832892.pdf
Advertising	Flyers_06162023.pdf
Advertising	Flyers_06162023_Highlighted.pdf
Advertising	Mailer OASIS .pdf

Research Procedures

Describe how the research will be conducted.

- What experience will study participants have?
- What will study participants be expected to do?
- How long will the study last?
- Outline the schedule and timing of study procedures.
- Provide visit-by-visit listing of all procedures that will take place.
- Identify all procedures that will be carried out with each group of participants.
- Describe deception and debrief procedures if deception is involved.

Differentiate between procedures that involve standard/routine clinical care and those that will be performed specifically for this research project. List medications that are explicitly forbidden or permitted during study participation.

There will be two parts to this study that are happening concurrently. Stakeholders will be convening at three timepoints: at study start-up, at study midpoint, and again at the end of the study. While this is happening, peer coaches and peer participants will also be participating in the intervention. See below the procedures each group will follow.

Study Stakeholders
At study start-up, we will recruit study stakeholders (community leaders, older adults with T2D, representatives from BRADD organizations [Aging on Aging case worker, Community Action], etc), obtain informed consent and conduct a series of 1–3 team meetings with them with the following goals: (1) present the final adapted version of the peer health coaching EBI to identify any possible final adaptations to promote pragmatic implementability; (2) finalize operationalization of RE-AIM dimensions based on shared implementation goals; and (3) identify potential facilitators and barriers that might influence implementation. To achieve the third goal, we will use an interview guide tested in previous applications of PRISM (see attachment) across health systems to analyze PRISM contextual determinants, such as external environment, implementation and sustainability infrastructure, and characteristics of participants of the EBI and of the EBI itself.

Midway through the intervention, we will again convene stakeholders for two additional meetings with three primary goals: (1) assess progress on RE-AIM dimensions; (2) select RE-AIM dimensions that require additional attention, if necessary; and (3) give context to understand the reasoning behind any differences that might exist between pre-implementation (i.e., anticipated) and midstream (i.e., unanticipated) adaptations. To achieve the first two goals, we will use an iterative approach to RE-AIM, wherein at the first meeting, team members will be reminded of RE-AIM dimensions selected at project start-up and then asked to confidentially rate the importance and progress of each RE-AIM dimension thus far on a 5-point Likert scale with options to provide qualitative explanatory feedback for each rating. At the second meeting, we will present de-identified results from the survey and then engage in subsequent brainstorming/goal setting to determine best approaches, including possible midcourse EBI adaptations, to improve data collection for RE-AIM dimensions deemed most in need of increased attention. To meet the third goal, we will use a PRISM-based survey (see attached, Pittman et al., 2021) and the same interview guide used at project start-up to give context to determinants of midcourse progress (including any identified adaptations).

Immediately upon program conclusion, we will convene a final team meeting with implementers and research team members, as well as a sample of randomly selected peer health coaches from each of the four factorial conditions, to collect RE-AIM outcomes data and conduct summative interviews with the same PRISM survey and qualitative interview guide as used at project onset and midpoint. Additionally, we will ask research team members, implementers, and selected peer health coaches to complete a psychometrically validated brief 12-item survey (see attached, Weiner et al., 2017) on perceptions of feasibility, acceptability, and appropriateness of both the overall EBI and each of the selected strategies (i.e., each condition in the 2x2 factorial design); we will also randomly select participants from each condition to complete the same survey and complete exit interviews to explore perceived and actual factors underscoring participant satisfaction with the EBI and its components.

The meetings with stakeholders will occur via Zoom or in-person based on availability of members.

Peer Participants
Eligible participants (meeting eligibility criteria and passing screening for no cognitive impairment) will be consented, enrolled and randomized into one of the groups.
1. Self-select mentor, once per week contact.
2. Self-select mentor, every two weeks contact
3. Matched with mentor, once per week contact.
4. Matched with mentor, every two weeks contact.

Participant-peer health coach matching. For participants who are randomized into a group where they are matched with a peer health coach, matching areas include similarity of life stage (e.g., age), county of residence, and duration of disease diagnosis. However, the primary matching area for this study will be county of residence. This decision was informed by key informants who highlighted that Appalachians' sense of identity is closely linked with the county in which they reside and that there will be an additional level of trust and comfort (or lack thereof) based on where their peer resides.

Participant self-selection of peer coach. For participants who are randomized into the group where they are able to self-select their peer coach, they will be provided with profiles of eligible coaches. These profiles will include age, sex, marital status, where they reside (county and town), duration of diabetes diagnosis, current HbA1c, and hobbies. Peer participants will be able to rank their top 3

coaches. The study team will try to match participants with their preferred coach. However, once a coach has been assigned 3 peer participants, they will be removed from the pool of coaches to choose from.

Peer coaches:
Coaches who are interested in the study will contact research team and be prescreened prior to enrollment. As part of the coach screening interviews with the PI, psychosocial status will be assessed described in Data Collection below. The PI will make a final determination of competence, maturity, emotional stability, and verbal communication skills after overall assessment during the screening interview and training. At this time, the research team will obtain consent. Each potential peer coach will undergo pre-intervention training, which is based off of "IDECIDE: Diabetes Peer Coaching Program Toolkit" developed by Dr. Michelle Heisler, and screened by stakeholders for cultural relevance. They will complete structured curriculum modules on working collaboratively with patients, basics of diabetes including self-care activities, knowledge of diabetes medications, recognizing medical "red flags" (e.g., symptoms of hypoglycemia), navigating the clinic, and assessing community resources. The peer coaches will also have training on informal skills development including active listening, non-judgmental communication, and positive social and emotional support (see peer coach training attachment).
Only coaches who pass a written and oral examination are included in the study. Oral examinations will be done by asking participants to preform mock calls with one another. Study personnel will observe and will score as either pass or fail.

Peer coaches and Peer participants

After peer participants are recruited, consented, and randomly assigned and peer coaches are recruited, consented, and trained, peer participants and coaches will attend an introductory session together, during which the coaching process will be discussed, including time commitment, roles, responsibilities, benefits, and ground rules, and the quads will have the opportunity to ask questions and make informed decisions about their ability to fully participate in the intervention. If face-to-face meetings are not possible for all members of the “quad,” phone or a form of video meeting will be attempted. At this time, the research coordinator will collect demographics, validated questionnaires, and baseline point-of-care clinical outcomes (Hemoglobin A1c via fingerstick). This introductory meeting as well as the peer coach-peer participant meetings during the 6 months of intervention can be in-person in community settings or via Zoom, depending upon participants/coach preference.

Intervention
During the intervention, peer coaches interact with the peer participant for 6 months, with the frequency of interactions based on whichever group the peer participant was randomly assigned to as described above. It is also optional for the peer coach to accompany the participant to at least one clinic visit. Topics to be discussed include current and target clinical goals for A1C, LDL, blood pressure, self-care activities, managing stress, and the SMART objectives.

Coach-peer interactions will be documented with the following information: date, type of encounter (phone, in-person), duration, and topics discussed.

Coaches will attend a monthly meeting to reinforce diabetes knowledge and communication skills. This meeting will be an open discussion based on their experiences interfacing with participants. We will use Zoom for the monthly meetings.

Peer participants will receive a link to a brief REDCap survey by email every two weeks. If internet access is limited, we will send a hardcopy to the participant with a self-addressed stamped envelope. This survey will assess the frequency and duration of calls, other interactions with their peer, and the specific content that was covered. These self-report assessments will be used to track the effectiveness of the intervention. A Modification request can be submitted later for this survey.

At the end of the intervention, the research coordinator will collect HbA1C via fingerstick and the validated questionnaire data described in data collection from peer coaches and peer participants.

Peer participants will then provide HbA1C levels and validated questionnaires again at 3- and 6-months post intervention.

There will be no PHI or health information obtained from or placed into participants' medical records.

Attachments

Attach Type	File Name
ResearchProcedures	PRISM.pdf
ResearchProcedures	Pittman et al., 2021.pdf
ResearchProcedures	Weiner et al., 2017.pdf
ResearchProcedures	PCTraining_01052023.pdf
ResearchProcedures	Peer Coach Competency Test.docx

Data Collection & Research Materials

In this section, please provide the following:

- Describe all sources or methods for obtaining research materials about or from living individuals (such as specimens, records, surveys, interviews, participant observation, etc.), and explain why this information is needed to conduct the study.
- For each source or method described, please list or attach all data to be collected (such as genetic information, interview scripts, survey tools, data collection forms for existing data, etc.).
- If you will conduct a record or chart review, list the beginning and end dates of the records you will view.

Peer participants and coaches will give blood via fingerstick to test hemoglobin A1c. Research staff will collect demographic characteristics (age, sex, race/ethnicity, marital status, county of residence, and income) from peer participants and coaches as well. Peer participants will have HgbA1c of greater or equal to 7.5 in the last 6 months and this will be verified by the participant and/or the person(s) who referred the peer participant.

Physical function will be assessed using Lawton's Instrumental Activities for Daily Living Scale, which evaluates the ability to independently use the toilet, feed, dress, groom, bathe and perform physical ambulation. We will measure frailty phenotype using a validated 14-item assessment that characterizes frailty by assessing usual state for mood, number of medications, mobility, function, balance, social connections, daytime tiredness, memory and thinking, vision, hearing, pain, unintentional weight-loss, aggression, and bladder control. Results will be scored on a 0 (no frailty, very fit) to 43 (severely frail) point scale. By identifying frailty phenotype, we can identify individuals at increased risk for adverse health outcomes, which serves as an indicator for complex patient management. Cognitive function will be measured using the Montreal Cognitive Assessment-Basic (MoCA-B) to ensure that study participants have the capacity to engage in study activities. Participants who are determined to have satisfactory physical and cognitive capabilities will be randomized.

They will complete validated questionnaires to measure social support (Medical Outcomes Study [MOS] Social Support Survey), medication adherence (Brooks Medication Adherence scale), quality of life (EuroQol-5D [EQ-5D]), self-care (Diabetes Self-Management Questionnaire [DSMQ]), problem solving skills (Diabetes Problem-Solving Inventory [DPSI]), empowerment (Diabetes Empowerment Scale), emotional distress (The Problem Areas in Diabetes Survey [PAID]), depressive symptoms (The Geriatric Depression Scale), diabetes knowledge (Diabetes Knowledge Questionnaire [DKQ]), smoking habits (Smoking Measures) as well as the LUBBEN SOCIAL NETWORK SCALE – 6 (LSNS-6).

Peer coaches. As part of the screening interview with the PI's, psychosocial status will be assessed using the psychological scales of the Wallston General Perceived Competence Scale, Campbell Personal Competence Index, Carkhuff Communication and Discrimination Skills Inventories, and the Applied Knowledge Assessment (AKA) scale (see https://onlinelibrary.wiley.com/doi/epdf/10.1002/art.1790060108). The PIs will make a determination of competence, maturity, emotional stability, and verbal communication skills after overall assessment during the screening interview and training.

Stakeholders. Interview data will be collected at study startup, midpoint intervention and at the end of intervention as discussed in research procedures.

Participants will complete all questionnaires using REDCap.

Attachments

Attach Type	File Name
DataCollection	OASIS_Demos .pdf
DataCollection	Smoking Measures.pdf
DataCollection	LUBBEN SOCIAL NETWORK SCALE Short form.pdf
DataCollection	MOCA-Basic.pdf
DataCollection	Diabetes Empowerment Scale.pdf
DataCollection	Diabetes Problem-Solving Inventory.pdf
DataCollection	EuroQofL-5D.pdf
DataCollection	Medical Outcomes Social Support Survey.pdf
DataCollection	Geriatric Depressive Scale Short Form.pdf
DataCollection	Problem Areas in Diabetes Survey.pdf
DataCollection	Diabetes Knowledge Questionnaire.pdf
DataCollection	Diabetes Self-Management Questionnaire.pdf
DataCollection	Brooks Medication Adherence.pdf
DataCollection	lawton& Brody-iadl.pdf
DataCollection	PFFS_v1.pdf

Resources

Describe the availability of the resources and adequacy of the facilities that you will use to perform the research. Such resources may include:

- Staffing and personnel, in terms of availability, number, expertise, and experience;
- Computer or other technological resources, mobile or otherwise, required or created during the conduct of the research;
- Psychological, social, or medical services, including equipment needed to protect subjects, medical monitoring, ancillary care, or counseling or social support services that may be required because of research participation;
- Resources for communication with subjects, such as language translation/interpretation services.

Dr. Brittany Smalls (health services and health equity researcher) is a mid-career minority investigator and PI of this study. She has extensive experience in conducted research on Appalachian KY participants and diabetes interventions.

Dr. Aaron Kruse-Diehr (implementation scientist) is a behavioral health researcher who has partnered with rural clinic and community

partners to assess outcomes related to implementation success, both within the context of large evidence-based colorectal cancer screening trials (UH3CA233282) and practice facilitation interventions to assist clinicians with referring patients with T2D to diabetes self-management education and support (DSMES) services (R34DK132548). Dr. Kruse-Diehr will lend his experience in applying implementation science frameworks and models to promote “real-world” success of interventions.

Dr. Key Douthitt (family medicine physician) has used his clinical expertise in other collaborative efforts alongside Dr. Smalls to alleviate health disparities, specifically concerning T2DM, in rural Appalachia Kentucky. For this study, his knowledge and experience as a clinician in rural Kentucky communities will be vital to participant recruitment, intervention administration and clinical monitoring of participant outcomes during the study.

Dr. Christopher McLouth (biostatistician) will use his expertise to determine preliminary clinical effectiveness of the intervention.

A UK computer will be used to store electronic data, which will be password protected and encrypted. Data will be stored and maintained behind a firewall on UK's network. The UK Computer will be locked in Suite 125 of the Department of Family and Community Medicine at Turfland. Only study staff members trained in human subjects protection will have permitted access to the data.

All paper files (informed consent) will be stored in a locked cabinet in the locked office of Dr. Smalls at UK until 6 years after study completion.

REDCap is implemented as a secure webserver (HTTPs) located within IPOP behind a firewall on UK's network. Accounts are created using the mc/ad domain account generated by UK and is used for authentication purposes. This login process requires individual password protection for investigators. Only research study personnel listed will have access to the data. The data will not be saved on the server to ensure confidentiality. Data entered on computer files will be password protected. The data collected and analyzed for this study will be specific to the project and not used for any other research purposes unless approved by the IRB. Data will be analyzed as a whole and no individual names will be included in reports.

Potential Risks & Benefits

Risks

- Describe any potential risks – including physical, psychological, social, legal, ability to re-identify subjects, or other risks. Assess the seriousness and likelihood of each risk.
- Which risks may affect a subject’s willingness to participate in the study?
- Describe likely adverse effects of drugs, biologics, devices or procedures participants may encounter while in the study.
- *Qualitative research* - describe ethical issues that could arise while conducting research in the field and strategies you may use to handle those situations.
- Describe any steps to mitigate these risks.

Benefits

- Describe potential direct benefits to study participants – including diagnostic or therapeutic, physical, psychological or emotional, learning benefits. This cannot include incentives or payments.
- State if there are no direct benefits.
- Describe potential benefits to society and/or general knowledge to be gained.

Describe why potential benefits are reasonable in relation to potential risks. If applicable, justify why risks to vulnerable subjects are reasonable to potential benefits.

Potential risks from participating in this study include a breach of confidentiality, and possible emotional distress when discussing your self-management of Type 2 Diabetes and goals with coaches and other peers in their sessions. The risks of taking blood via fingerstick include pain, a bruise at the point where the blood is taken, redness and swelling, and a rare risk of fainting. In addition, stakeholders could experience emotional distress when providing feedback regarding the intervention, especially if they have a loved one suffering from type 2 diabetes.
Benefits to the participant include a high level of satisfaction in their ability to manage their diabetes and improved clinical outcomes from participating in the weekly sessions with their coach, and helping others manage type 2 diabetes

Available Alternative Opportunities/Treatments

Describe alternative treatments or opportunities that might be available to those who choose not to participate in the study, and which offer the subject equal or greater advantages. If applicable, this should include a discussion of the current standard of care treatment(s).

There are no alternative treatments available to the stakeholders and peer coaches if they choose not to participate in this study. Peer participants can seek out their own peer coaches outside of the study if they choose to do so.

Back to Top

Records, Privacy, and Confidentiality

Specify where the data and/or specimens will be stored and how the researcher will ensure the privacy and confidentiality of both. Specify who will have access to the data/specimens and why they need access.

Describe how data will be managed after the study is complete:

- If data/specimens will be maintained, specify whether identifiers will be removed from the maintained information/material.
- If identifiers will not be removed, provide justification for retaining them and describe how you will protect confidentiality.
- If the data/specimens will be destroyed, verify that this will not violate retention policies and will adhere to applicable facility requirements.

If this study will use de-identified data from another source, describe what measures will be taken to ensure that subject identifiers are not given to the investigator.

If applicable, describe procedures for sharing data/specimens with collaborators not affiliated with UK.

For additional considerations:
Return of Research Results or Incidental Research Findings
HIPAA policies
FERPA policies
Procedures for Transfer agreements
Information regarding multi-site studies
NIH Genomic Data Sharing (GDS) Policy
Digital Data

Demographic data to be collected at the beginning of the study includes age, sex, race/ethnicity, marital status, county of residence, and income. Physical function will be assessed with Lawton's Instrumental Activities for Daily Living Scale which evaluates the ability to independently use the toilet, feed, dress, groom, bathe and perform physical ambulation. Cognitive Function will be assessed using the Montreal Cognitive Assessment-Basic (MoCA-B) to ensure that study participants have the capacity to engage in study activities. Those who are determined to have satisfactory physical and cognitive capabilities will be randomized.

Frailty: We will measure frailty phenotype using a validated 4-item assessment that characterizes frailty by exhaustion, low physical activity, weakness, and low body mass index. Individuals are characterized as frail if they have affirmative responses to 3 or 4 of the items. By identifying frailty phenotype, we can identify individuals at increased risk for adverse health outcomes, which serves as an indicator for complex patient management.

Data will be collected from participants at the beginning of the study, as well as at 6-month (end of intervention) to assess the following:
Social Support: Medical Outcomes Study (MOS) Social Support Survey uses a 5-point Likert scale response to their perceived social support with a corresponding value to assess perceived social support.
Quality of Life: EuroQol-5D (EQ-5D) is validated measure of 5 dimensions of health-related quality of life including mobility, self-care, usual activities, pain/discomfort, and anxiety/depression. The EQ-5D also includes a Visual Analogue Scale by which respondents can report their perceived health status ranging from 0 (the worst possible health) to 100 (the best possible health).
Self-Care: The Diabetes Self-Management Questionnaire (DSMQ) is a 16-item questionnaire to assess self-care activities activity associated with glycemic control and has an internal consistency of 0.84.
Problem solving skills: The Diabetes Problem-Solving Inventory (DPSI) includes 9-items that assess how individuals living with diabetes cope with challenges of T2DM-related self-care. The inventory measures three components—healthy eating, physical activity, and stress management. Overall problem-solving rating is provided on a 5-point scale from 1 (very poor strategy) to 5 (excellent strategy).
Empowerment: The Diabetes Empowerment Scale is a 23-item scale that measures diabetes-related psychosocial self-efficacy with an overall Cronbach's a of 0.96 uses 3 subscales: Managing the Psychosocial Aspects of Diabetes, Assessing Dissatisfaction and Readiness to Change, and Setting and Achieving Diabetes Goals.
Diabetes Knowledge: Diabetes Knowledge Questionnaire (DKQ) a 24-item survey that has a reliability coefficient of 0.78 and showed sensitivity to a diabetes knowledge intervention. In addition, it targets knowledge deficits which can be related to measurable outcomes, false statements or those known to be common and/or serious misconceptions.
Clinical outcomes: hemoglobin A1c
Medication Adherence: The Brooks Medication Adherence Scale is a 6-item scale with a reliability coefficient >0.69 and is sensitive to changes in adherence due to intervention.
Emotional distress: The Problem Areas in Diabetes Survey (PAID) includes 20 items designed to assess diabetes-related emotional distress among adults. The questions are answered using a 6-point Likert scale from 1 (no problem) to 6 (serious problem). The PAID has a Cronbach's alpha of 0.95.
Depressive symptoms: The Geriatric Depression Scale is a screening measure used in the elderly that is sensitive to those suffering from mild cognitive impairment and physical illness.
All data will be de-identified and stored on a password-protected computer at the University of Kentucky, including health information. In order to track participation over time, we will use an enrollment log that includes participants' study IDs but will only use study IDs (e.g., de-identify) when entering data into RedCap. Only authorized individuals will have access to study data.

All precautions will be taken to keep participants safe. To secure against a potential breach of confidentiality, all study files will be deidentified and kept on a password-protected secure server at the University of Kentucky. All hard copies of research documents will be stored in a locked cabinet in Dr. Small's office space. Research documents including participant identifying information (i.e., informed consent documents, signed receipts for participant payments) will be stored separately from data documents.

Based on the availability and preferences of stakeholders we will have in-person interviews that will be audio-recorded or we may have

videoconference interviews where stakeholders will be given the option of using a camera or not. In the case of videoconference interviews, the interview will be audio-recorded even if the video function is not used. The video transcripts will remain part of study data for a minimum of 6 years post data collection. All stakeholder interviews are considered data and will be secured in an online server that is password protected and only accessible to IRB approved personnel.

All interview data with stakeholders (interview notes, audio and video (if applicable) recordings, and transcriptions of audiotapes) will be coded using participant ID numbers rather than names or other identifying information. An electronic link file of participant IDs and names will be maintained by the study coordinator in a password protected file stored on a secure server housed at the University of Kentucky. Should any transfer of electronic files between research team members occur, the transfer would be completed using the encrypted file transfer function "Send It" available through the REDCap platform supported by CCTS. All electronic files regardless of identifiers will be password protected and accessible only by IRB approved and human subjects protection-trained study staff. At the conclusion of each interview, the audio recording will be uploaded to an encrypted UK laptop computer, saved and password protected on a secure UK server, named using an assigned ID number, deidentified by a trained member of the research team, and stored with no connection to the password protected link file. Audio recordings will be deleted from the recording devices as soon as uploading is verified and complete. Transcripts of the audio recordings will be named and stored securely in the same way, with any spoken names and identifying information redacted from the typed content of the interview. Hard copies of transcripts of interviews will be labeled only with assigned ID numbers and stored in a locked file cabinet in Dr. Kruse-Diehr's office in The Department of Family and Community Medicine, Suite 125 Turfland. Again, only study staff members who are trained in human subjects protection will be allowed to access data. At the completion of the study, all research documents (including consent forms, questionnaires, audio-recordings, and electronic and hard copies of transcripts) will be stored securely electronically (as described above) or in a locked cabinet for 6 years after the end of the project, depending on whether the document is in a hard copy or electronic copy format.

UK IRB policies state that IRB-related research records must be retained for a minimum of 6 years after study closure. Do you confirm that you will retain all IRB-related records for a minimum of 6 years after study closure?

(•) Yes ( ) No

Payment

Describe the incentives (monetary or other) being offered to subjects for their participation. If monetary compensation is offered, indicate the amount and describe the terms and schedule of payment. Please review this guidance for more information on payments to subjects, including restrictions and expectations.

For the peer coaching intervention, coaches will be compensated $150 for completing pre-intervention peer coach training. They will also be compensated $25/month for conducting weekly check-in sessions with up to three peers for 6 months. Payment will be provided as physical gift cards and will be received in person and/or email. Coaches will not receive incentives for providing A1C and completing validated surveys at baseline and at 6 months (end of intervention). Thus, coaches can be paid a total of $300 for participating.

Peer participants will be compensated $20 for completing assessments at baseline, 6 months (end of intervention), and at both 3 and 6-month follow up data collection for a total of $80. Payment will be provided as physical gift cards and will be received in person and/or email.

Stakeholders will not receive compensation for participation.

Costs to Subjects

Include a list of services and/or tests that will not be paid for by the sponsor and/or the study (e.g., MRI, HIV). Keep in mind that a subject will not know what is “standard” – and thus not covered by the sponsor/study – unless you tell them.

There are no costs to subjects other than the time required to complete surveys and attend coaching sessions.

Data and Safety Monitoring

The IRB requires review and approval of data and safety monitoring plans for greater than minimal risk research or NIH-funded/FDA-regulated clinical investigations.

- If you are conducting greater than minimal risk research, or your clinical investigation is NIH-funded, describe your Data and Safety Monitoring Plan (DSMP). Click here for additional guidance on developing a Data and Safety Monitoring Plan.
- If this is a non-sponsored investigator-initiated protocol considered greater than minimal risk research, and if you are planning on using a Data and Safety Monitoring Board (DSMB) as part of your DSMP, click here for additional guidance for information to include with your IRB application.

DSMP is in "Additional information/Materials"

Back to Top

Future Use and Sharing of Material (e.g., Data/Specimens/Information)

If the material collected for this study will be used by members of the research team or shared with other researchers for future studies, please address the following:

- list the biological specimens and/or information that will be kept
- briefly describe the types, categories and/or purposes of the future research
- describe any risks of the additional use
- describe privacy/confidentiality protections that will be put into place
- describe the period of time specimens/information may be used
- describe procedures for sharing specimens/information with secondary researchers
- describe the process for, and limitations to, withdrawal of specimens/data

Information on demographic characteristics (age, sex, race/ethnicity, marital status, county of residence, income), social support, self-care, medication adherence, problem-solving skills, emotional distress, diabetes knowledge, empowerment, depressive symptoms, quality of life, baseline point-of-care clinical outcomes, physical function, and frailty will be collected and stored. All precautions will be taken to keep participants safe. To secure against a potential breach of confidentiality, all data will be de-identified and stored on a password-protected computer at the University of Kentucky, including health information. In order to track participation over time, we will use an enrollment log that includes participants' study IDs but will only use study IDs (e.g., deidentify) when entering data into RedCap. Only authorized individuals will have access to study data. Data and electronic records will be kept for a minimum of six years post-study closure. Your information or samples collected for this study will NOT be used or shared for future research studies, even if we remove the identifiable information like your name, medical record number, or date of birth.

Are you recruiting or expect to enroll **Non-English Speaking Subjects or Subjects from a Foreign Culture**? (does not include short form use for incidentally encountered non-English subjects)

○ Yes ◉ No

Non-English Speaking Subjects or Subjects from a Foreign Culture

Recruitment and Consent:

Describe how information about the study will be communicated to potential subjects appropriate for their culture, and if necessary, how new information about the research may be relayed to subjects during the study.
When recruiting Non-English-speaking subjects, provide a consent document in the subject's primary language. After saving this section, attach both the English and translated consent documents in the "Informed Consent" section.

Cultural and Language Consultants:

The PI is required to identify someone who is willing to serve as the cultural consultant to the IRB.

- This person should be familiar with the culture of the subject population and/or be able to verify that translated documents are the equivalent of the English version of documents submitted.
- The consultant should not be involved with the study or have any interest in its IRB approval.
- Please include the name, address, telephone number, and email of the person who agrees to be the cultural consultant for your study.
- ORI staff will facilitate the review process with your consultant. Please do not ask them to review your protocol separately.

For more details, see the IRB Application Instructions on Research Involving Non-English Speaking Subjects or Subjects from a Foreign Culture.

Local Requirements:

If you will conduct research at an international location, identify and describe:

- relevant local regulations
- data privacy regulations
- applicable laws
- ethics review requirements for human subject protection

Please provide links or sources where possible. If the project has been or will be reviewed by a local ethics review board, attach a copy in the "Additional Information/Materials" section. You may also consult the current edition of the International Compilation of Human Research Standards

Does your study involve **HIV/AIDS research and/or screening for other reportable diseases (e.g., Hepatitis C, etc…)**?
○ Yes ◉ No

HIV/AIDS Research

If you have questions about what constitutes a reportable disease and/or condition in the state of Kentucky, see ORI's summary sheet: "Reporting Requirements for Diseases and Conditions in Kentucky" [PDF].

HIV/AIDS Research: There are additional IRB requirements for designing and implementing the research and for obtaining informed consent. Describe additional safeguards to minimize risk to subjects in the space provided below.

For additional information, visit the online IRB Survival Handbook to download a copy of the "Medical IRB's requirements for Protection of Human Subjects in Research Involving HIV Testing" [D65.0000] [PDF], and visit the Office for Human Research Protections web site for statements on AIDS research, or contact the Office of Research Integrity at 859-257-9428.

PI-Sponsored FDA-Regulated Research

Is this an investigator-initiated study that:

1) involves testing a Nonsignificant Risk (NSR) Device, or
2) is being conducted under an investigator-held Investigational New Drug (IND) or Investigational Device Exemption (IDE)?
○ Yes ◉ No

PI-Sponsored FDA-Regulated Research

If the answer above is yes, then the investigator assumes the regulatory responsibilities of both the investigator and sponsor. The Office of Research Integrity provides a summary list of sponsor IND regulatory requirements for drug trials [PDF], IDE regulatory requirements for SR device trials [PDF], and abbreviated regulatory requirements for NSR device trials [PDF]. For detailed descriptions see FDA Responsibilities for Device Study Sponsors or FDA Responsibilities for IND Drug Study Sponsor-Investigators.

- Describe the experience/knowledge/training (if any) of the investigator serving as a sponsor (e.g., previously held an IND/IDE); and
- Indicate if any sponsor obligations have been transferred to a commercial sponsor, contract research organization (CRO), contract monitor, or other entity (provide details or attach FDA 1571).

IRB policy requires mandatory training for all investigators who are also FDA-regulated sponsors (see Sponsor-Investigator FAQs). A sponsor-investigator must complete the applicable Office of Research Integrity web based training, (drug or device) before final IRB approval is granted.

Has the sponsor-investigator completed the mandatory PI-sponsor training prior to this submission?
○ Yes ○ No

If the sponsor-investigator has completed equivalent sponsor-investigator training, submit documentation of the content for the IRB's consideration.

Attachments

HIPAA

0 unresolved comment(s)

Is HIPAA applicable? ○ Yes ◉ No

(Visit ORI's Health Insurance Portability and Accountability Act (HIPAA) web page to determine if your research falls under the HIPAA Privacy Regulation.)

If yes, check below all that apply and attach the applicable document(s):

- ☐ HIPAA De-identification Certification Form
- ☐ HIPAA Waiver of Authorization

Attachments

STUDY DRUG INFORMATION

0 unresolved comment(s)

The term drug may include:

- FDA approved drugs,
- unapproved use of approved drugs,
- investigational drugs or biologics,
- other compounds or products intended to affect structure or function of the body, and/or
- complementary and alternative medicine products such as dietary supplements, substances generally recognized as safe (GRAS) when used to diagnose, cure mitigate, treat or prevent disease, or clinical studies of e-cigarettes examining a potential therapeutic purpose.

Does this protocol involve a drug including an FDA approved drug; unapproved use of an FDA approved drug; and/or an investigational drug?

○ Yes ◉ No

If yes, complete the questions below. Additional study drug guidance.

LIST EACH DRUG INVOLVED IN STUDY IN THE SPACE BELOW

Drug Name:

Note: Inpatient studies are required by Hospital Policy to utilize Investigational Drug Service (IDS) pharmacies (Oncology or Non-Oncology). Use of IDS is highly recommended, but optional for outpatient studies. Outpatient studies not using IDS services are subject to periodic inspection by the IDS for compliance with drug accountability good clinical practices.

Indicate where study drug(s) will be housed and managed:

☐ Investigational Drug Service (IDS) UK Hospital

Other Location:

Is the study being conducted under a valid Investigational New Drug (IND) application?

○ Yes ○ No

If Yes, list IND #(s) and complete the following:

IND Submitted/Held by:

Sponsor: ☐ Held By:

Investigator: ☐ Held By:

Other: ☐ Held By:

☐ Checkmark if the study is being conducted under FDA's Expanded Access Program (e.g., Treatment IND) or if this is an Individual Patient Expanded Access IND (FDA Form 3926).

FDA's Expanded Access Program Information for Individual Patient Expanded Access INDs, and attach the following:

- FDA Form 3926;
- FDA expanded access approval or correspondence;
- Confirmation of agreement from manufacturer or entity authorized to provide access to the product.

For guidance and reporting requirements at the conclusion of treatment see the Expanded Access SOP.

Complete and attach the required Study Drug Form picking "Study Drug Form" for the document type. Any

applicable drug documentation (e.g., Investigator Brochure; approved labeling; publication; FDA correspondence, etc.) should be attached using "Other Drug Documentation" for the document type.

Attachments

STUDY DEVICE INFORMATION

0 unresolved comment(s)

A DEVICE may be a:

- component, part, accessory;
- assay, reagent, or in-vitro diagnostic device;
- software, digital health, or mobile medical app;
- other instrument if intended to affect the structure or function of the body, diagnose, cure, mitigate, treat or prevent disease; or
- a homemade device developed by an investigator or other non-commercial entity and not approved for marketing by FDA.

For additional information, helpful resources, and definitions, see ORI's Use of Any Device Being Tested in Research web page.

Does this protocol involve testing (collecting safety or efficacy data) of a medical device including an FDA approved device, unapproved use of an approved device, humanitarian use device, and/or an investigational device?

( ) Yes (•) No

[Note: If a marketed device(s) is only being used to elicit or measure a physiologic response or clinical outcome, AND, NO data will be collected on or about the device itself, you may answer "no" above, save and exit this section, (Examples: a chemo drug study uses an MRI to measure tumor growth but does NOT assess how effective the MRI is at making the measurement; an exercise study uses a heart monitor to measure athletic performance but no safety or efficacy information will be collected about the device itself, nor will the data collected be used for comparative purposes against any other similar device).]

If you answered yes above, please complete the following questions.

LIST EACH DEVICE BEING TESTED IN STUDY IN THE SPACE BELOW

Device Name:

Is the study being conducted under a valid Investigational Device Exemption (IDE), Humanitarian Device Exemption (HDE) or Compassionate Use?

( ) Yes ( ) No

If Yes, complete the following:
IDE or HDE #(s)

IDE/HDE Submitted/Held by:

Sponsor: [ ]	Held By:
Investigator: [ ]	Held By:
Other: [ ]	Held By:

[ ] Check if this is a Treatment IDE or Compassionate Use under the Food and Drug Administration (FDA) Expanded Access program.

For Individual or Small Group Expanded Access, see FDA's Early Expanded Access Program Information, and attach the following:

- FDA expanded access approval or sponsor's authorization;
- An independent assessment from an uninvolved physician, if available;
- Confirmation of agreement from manufacturer or entity authorized to provide access to the product.

For guidance and reporting requirements at the conclusion of treatment see the Medical Device SOP.

Does the intended use of any research device being tested (not clinically observed) in this study meet the regulatory definition of Significant Risk (SR) device?

- Yes. Device(s) being tested in this study presents a potential for serious risk to the health, safety, or welfare of a subject and (1) is intended as an implant; or (2) is used in supporting or sustaining human life; or (3) is of substantial importance in diagnosing, curing, mitigating or treating disease, or otherwise prevents impairment of human health; or (4) otherwise presents a potential for serious risk to the health, safety, or welfare of a subject.
- No. All devices being tested in this study do not present a potential for serious risk to the health, safety, or welfare of subjects/participants.

Complete and attach the required Study Device Form, picking the "Study Device Form" for the document type. Any applicable device documentation (e.g., Manufacturer information; patient information packet; approved labeling; FDA correspondence, etc.) should be attached using "Other Device Documentation" for the document type.

Attachments

RESEARCH SITES

0 unresolved comment(s)

To complete this section, ensure the responses are accurate then click "SAVE".

A) Check all the applicable sites listed below at which the research will be conducted. If none apply, you do not need to check any boxes.

UK Sites

- [ ] UK Classroom(s)/Lab(s)
- [ ] UK Clinics in Lexington
- [x] UK Clinics outside of Lexington
- [ ] UK Healthcare Good Samaritan Hospital
- [ ] UK Hospital

Schools/Education Institutions

- [ ] Fayette Co. School Systems *
- [ ] Other State/Regional School Systems
- [ ] Institutions of Higher Education (other than UK)

***Fayette Co. School systems, as well as other non-UK sites, have additional requirements that must be addressed. See ORI's IRB Application Instructions - Off-site Research web page for details.**

Other Medical Facilities

- [ ] Bluegrass Regional Mental Health Retardation Board
- [ ] Cardinal Hill Hospital
- [ ] Eastern State Hospital
- [ ] Norton Healthcare
- [ ] Nursing Homes
- [ ] Shriner's Children's Hospital
- [ ] Veterans Affairs Medical Center
- [ ] Other Hospitals and Med. Centers

- [ ] Correctional Facilities
- [ ] Home Health Agencies
- [ ] International Sites

Research activities conducted at performance sites that are not owned or operated by the University of Kentucky (UK) or at sites that do not fall under the UK IRB's authority, are subject to special procedures for coordination of research review. Additional information is required (see IRB Application Instructions - Off-Site Research web page), including:

- A letter of support and local context is required from non-UK sites. See *Letters of Support and Local Context* on the IRB Application Instructions - Off-Site Research web page for more information.
- Supportive documentation, including letters of support, can be attached below.
- NOTE: If the non-UK sites or non-UK personnel are engaged in the research, there are additional federal and university requirements which need to be completed for their participation. For instance, the other site(s) may need to complete their own IRB review, or a cooperative review arrangement may need to be established with non-UK sites.

- Questions about the participation of non-UK sites/personnel should be discussed with the ORI staff at (859) 257-9428.

List all other non-UK owned/operated locations where the research will be conducted:

Community settings(to be determined by participants/coaches whether they meet via zoom or in person in a community setting) - these will be listed here with appropriate documentation prior to beginning research in these locations.

Describe the role of any non-UK site(s) or non-UK personnel who will be participating in your research.

BRADD and Community Action of Southern KY will be referring participants to the study personnel

Attachments

Attach Type	File Name
-Letter of Support & Local Context	BSOASISBRADD.pdf
-Letter of Support & Local Context	BSOASISCommunity Action of Southern KY.pdf

B) Is this a multi-site study for which **you are the lead investigator or UK is the lead site**? ○ Yes ◉ No

If YES, describe the plan for the management of reporting unanticipated problems, noncompliance, and submission of protocol modifications and interim results from the non-UK sites:

C) If your research involves collaboration with any sites and/or personnel outside the University of Kentucky, then it is considered multisite research and IRB reliance issues will need to be addressed. This may include national multi-center trials as well local studies involving sites/personnel external to UK. If you would like to request that the University of Kentucky IRB (UK IRB) serve as the lead IRB for your study, or if you would like the UK IRB to defer review to another IRB, please contact the IRBReliance@uky.edu.

RESEARCH ATTRIBUTES

0 unresolved comment(s)

Indicate the items below that apply to your research. Depending on the items applicable to your research, you may be required to complete additional forms or meet additional requirements. Contact the ORI (859-257-9428) if you have questions about additional requirements.

- [ ] Not applicable

Check All That Apply

- [ ] Academic Degree/Required Research
- [ ] Alcohol/Drug/Substance Abuse Research
- [ ] Biological Specimen Bank Creation (for sharing)
- [ ] Cancer Research
- [ ] CCTS-Center for Clinical & Translational Science
- [ ] Certificate of Confidentiality
- [ ] Clinical Research
- [ ] Clinical Trial - Phase 1
- [ ] Clinical Trial
- [ ] Collection of Biological Specimens for internal banking and use (not sharing)
- [x] Community-Based Participatory Research
- [ ] Deception
- [ ] Educational/Student Records (e.g., GPA, test scores)
- [ ] Emergency Use (Single Patient)
- [ ] Gene Transfer
- [ ] Genetic Research
- [ ] GWAS (Genome-Wide Association Study) or NIH Genomic Data Sharing (GDS)
- [ ] Human Cells, Tissues, and Cellular and Tissue Based Products
- [ ] Individual Expanded Access or Compassionate Use
- [ ] International Research
- [ ] Planned Emergency Research Involving Exception from Informed Consent
- [ ] Recombinant DNA
- [ ] Registry or data repository creation
- [ ] Stem Cell Research
- [ ] Suicide Ideation or Behavior Research
- [x] Survey Research
- [ ] Transplants
- [ ] Use, storage and disposal of radioactive material and radiation producing devices
- [ ] Vaccine Trials

For additional requirements and information:

- Cancer Research (MCC PRMC)
- Certificate of Confidentiality (look up "Confidentiality/Privacy...")
- CCTS (Center for Clinical and Translational Science)
- Clinical Research (look up "What is the definition of....)
- Clinical Trial
- Collection of Biological Specimens for Banking (look up "Specimen/Tissue Collection...")
- Collection of Biological Specimens (look up "Specimen/Tissue Collection...")
- Community-Based Participatory Research (look up "Community-Engaged...")
- Data & Safety Monitoring Board (DSMB)

  *For Medical IRB: Service Request Form for CCTS DSMB

- Data & Safety Monitoring Plan
- Deception*

  *For deception research, also go to the E-IRB Application Informed Consent section, checkmark and complete "Request for Waiver of Informed Consent Process"

- Emergency Use (Single Patient) [attach Emergency Use Checklist] (PDF)
- Genetic Research (look up "Specimen/Tissue Collection...")
- Gene Transfer
- HIV/AIDS Research (look up "Reportable Diseases/Conditions")
- Screening for Reportable Diseases [E2.0000] (PDF)
- International Research (look up "International & Non-English Speaking")
- NIH Genomic Data Sharing (GDS) Policy (PDF)
- Planned Emergency Research Involving Waiver of Informed Consent*

  *For Planned Emergency Research Involving Waiver of Informed Consent, also go to the E-IRB Application Informed Consent section, checkmark and complete "Request for Waiver of Informed Consent Process"

- Use, storage and disposal of radioactive material and radiation producing devices

FUNDING/SUPPORT

0 unresolved comment(s)

If the research is being submitted to, supported by, or conducted in cooperation with an external or internal agency or funding program, indicate below all the categories that apply.

☐ Not applicable

Check All That Apply

- ☐ Grant application pending
- ☑ (HHS) Dept. of Health & Human Services
  - ☑ (NIH) National Institutes of Health
  - ☐ (CDC) Centers for Disease Control & Prevention
  - ☐ (HRSA) Health Resources and Services Administration
  - ☐ (SAMHSA) Substance Abuse and Mental Health Services Administration
- ☐ (DoJ) Department of Justice or Bureau of Prisons
- ☐ (DoE) Department of Energy
- ☐ (EPA) Environmental Protection Agency
- ☐ Federal Agencies Other Than Those Listed Here
- ☐ Industry (Other than Pharmaceutical Companies)
- ☐ Internal Grant Program w/ proposal
- ☐ Internal Grant Program w/o proposal
- ☐ National Science Foundation
- ☐ Other Institutions of Higher Education
- ☐ Pharmaceutical Company
- ☐ Private Foundation/Association
- ☐ U.S. Department of Education
- ☐ State

Other:

Click applicable listing(s) for additional requirements and information:

- (HHS) Dept. of Health & Human Services
- (NIH) National Institutes of Health
- (CDC) Centers for Disease Control & Prevention
- (HRSA) Health Resources & Services Administration
- (SAMHSA) Substance Abuse & Mental Health Services Administration
- Industry (Other than Pharmaceutical Companies) [IRB Fee Info]
- National Science Foundation
- (DoEd) U.S. Department of Education
- (DoJ) Department of Justice or Bureau of Prisons
- (DoE) Department of Energy Summary and Department of Energy Identifiable Information Compliance Checklist
- (EPA) Environmental Protection Agency

Specify the funding source and/or cooperating organization(s) (e.g., National Cancer Institute, Ford Foundation, Eli Lilly & Company, South Western Oncology Group, Bureau of Prisons, etc.):

National Institute of Diabetes and Digestive and Kidney Diseases

Add Related Grants

If applicable, please search for and select the OSPA Account number or Electronic Internal Approval Form (eIAF) # (notif #) associated with this IRB application using the "Add Related Grants" button.
If required by your funding agency, upload your grant using the "Grant/Contract Attachments" button.

Add Related Grants

Grant/Contract Attachments

The research involves use of Department of Defense (DoD) funding, military personnel, DoD facilities, or other DoD resources. (See DoD SOP and DoD Summary for details)

○ Yes ◉ No

Using the "attachments" button (below), attach applicable materials addressing the specific processes described in the DoD SOP.

DOD SOP Attachments

Additional Certification: (If your project is federally funded, your funding agency may request an Assurance/ Certification/Declaration of Exemption form.) Check the following if needed:

☐ Protection of Human Subjects Assurance/Certification/Declaration of Exemption (Formerly Optional Form – 310)

Assurance/Certification Attachments

OTHER REVIEW COMMITTEES

0 unresolved comment(s)

If you check any of the below committees, additional materials may be required with your application submission.

Does your research fall under the purview of any of the other review committees listed below? [*If yes, check all that apply and attach applicable materials using the attachment button at the bottom of your screen.*]

○ Yes ◉ No

Additional Information

- ☐ Institutional Biosafety Committee
- ☐ Radiation Safety Committee
- ☐ Radioactive Drug Research Committee
- ☐ Markey Cancer Center (MCC) Protocol Review and Monitoring Committee (PRMC)
- ☐ Graduate Medical Education Committee (GME)
- ☐ Office of Medical Education (OME)

- Institutional Biosafety Committee (IBC) - Attach required IBC materials
- Radiation Safety Committee (RSC) - For applicability, see instructions and attach form
- Radioactive Drug Research Committee (RDRC)
- Markey Cancer Center (MCC) Protocol Review and Monitoring Committee (PRMC)** - Attach MCC PRMC materials, if any, per instructions.
- Office of Medical Education (OME)
- Graduate Medical Education Committee (GME)

Attachments

**** If your study involves cancer research, be sure to select "Cancer Research" in the "Research Attributes" section.** ORI will send your research protocol to the Markey Cancer Center (MCC) Protocol Review and Monitoring Committee (PRMC). The MCC PRMC is responsible for determining whether the study meets the National Cancer Institute (NCI) definition of a clinical trial and for issuing documentation to you (the investigator) which confirms either that PRMC approval has been obtained or that PRMC review is not required. Your IRB application will be processed and reviewed independently from the PRMC review.

ADDITIONAL INFORMATION/MATERIALS

0 unresolved comment(s)

Do you want specific information inserted into your approval letter? ○ Yes ◉ No

Approval Letter Details:

If you wish to have specific language included in your approval letter (e.g., serial #, internal tracking identifier, etc…), type that language in the box below exactly as it should appear in the letter. The text you enter will automatically appear at the top of all approval letters, identical to how you typed it, until you update it. Don't include instructions or questions to ORI staff as those will appear in your approval letter. **If these details need to be changed for any reason, you are responsible for updating the content of this field.**

Additional Materials:

If you have other materials you would like to include for the IRB's consideration, check all that apply and attach the corresponding documents using the Attachments button below.

- ☐ Detailed protocol
- ☐ Dept. of Health & Human Services (DHHS) approved protocol (such as NIH sponsored Cooperative Group Clinical Trial)
- ☑ Other Documents

Protocol/Other Attachments

Attach Type	File Name
Other	E-IRB 83904 1st Set of Comments Stakeholder CF.pdf
Other	E-IRB 83904 1st Set of Comments Peer Coach Consent Form.pdf
Other	E-IRB 83904 1st Set of Comments PEER Consent Form.pdf
Other	OASIS DSMP__1 13 23.pdf

NOTE: Instructions for Dept. of Health & Human Services (DHHS)-approved protocol]

If you have password protected documents, that feature should be disabled prior to uploading to ensure access for IRB review.

To view the materials currently attached to your application, click “All Attachments” on the left menu bar.

SIGNATURES (ASSURANCES)

0 unresolved comment(s)

Introduction

All IRB applications require additional assurances by a Department Chairperson or equivalent (DA), and when applicable, a Faculty Advisor or equivalent (FA). This signifies the acceptance of certain responsibilities and that the science is meritorious and deserving of conduct in humans. The person assigned as DA *should not* also be listed in the Study Personnel section, and the individual assigned as FA *should* be listed in the Study Personnel section.

For a list of responsibilities reflected by signing the Assurance Statement, refer to "What does the Department Chairperson's Assurance Statement on the IRB application mean?"

For a detailed illustration of how to complete this section, please review the short online video tutorial "Signatures (Assurance) Section - How to Complete." Otherwise, follow the steps below.

Required Signatures:

First Name	Last Name	Role	Department	Date Signed	
Ginny	Gottschalk	Department Authorization	Family and Community Medicine	12/15/2022 12:06 PM	View/Sign
Brittany	Smalls	Principal Investigator	Family and Community Medicine	01/03/2023 02:41 PM	View/Sign

Department Authorization

☑ This is to certify that I have reviewed this research protocol and that I attest to the scientific validity and importance of this study; to the qualifications of the investigator(s) to conduct the project and their time available for the project; that facilities, equipment, and personnel are adequate to conduct the research; and that continued guidance will be provided as appropriate. When the principal investigator assumes a sponsor function, the investigator has been notified of the additional regulatory requirements of the sponsor and by signing the principal investigator Assurance Statement, confirms he/she can comply with them.

*If the Principal Investigator is also the Chairperson of the department, the Vice Chairperson or equivalent should complete the "Department Authorization".

**IF APPLICABLE FOR RELIANCE: I attest that the principal investigator has been notified of the regulatory requirements of both the Reviewing and Relying IRBs, according to the information provided in the E-IRB application. The attached Reliance Assurance Statement, signed by the principal investigator, confirms that he/she can comply with both sets of IRB requirements.

Principal Investigator's Assurance Statement

I understand the University of Kentucky's policies concerning research involving human subjects and I agree:

1. To comply with all IRB policies, decisions, conditions, and requirements;
2. To accept responsibility for the scientific and ethical conduct of this research study;
3. To obtain prior approval from the Institutional Review Board before amending or altering the research protocol or implementing changes in the approved consent/assent form;
4. To report to the IRB in accord with IRB/IBC policy, any adverse event(s) and/or unanticipated problem(s) involving risks to subjects;
5. To complete, on request by the IRB for Full and Expedited studies, the Continuation/Final Review Forms;
6. To notify the Office of Sponsored Projects Administration (OSPA) and/or the IRB (when applicable) of the development of any financial interest not already disclosed;
7. Each individual listed as study personnel in this application has received the mandatory human research protections

education (e.g., CITI);
8. Each individual listed as study personnel in this application possesses the necessary experience for conducting research activities in the role described for this research study.
9. To recognize and accept additional regulatory responsibilities if serving as both a sponsor and investigator for FDA regulated research.

☑ Furthermore, by checking this box, I also attest that:

- I have appropriate facilities and resources for conducting the study;
- I am aware of and take full responsibility for the accuracy of all materials submitted to the IRB for review;
- If applying for an exemption, I also certify that the only involvement of human subjects in this research study will be in the categories specified in the Protocol Type: Exemption Categories section.
- If applying for an Abbreviated Application (AA) to rely on an external IRB, I understand that certain items above (1, 3, 4, 7-8) may not apply, or may be altered due to external institutional/IRB policies. I document my agreement with the Principal Investigator Reliance Assurance Statement by digitally signing this application.

*You will be able to "sign" your assurance after you have sent your application for signatures (use Submission section). Please notify the personnel required for signing your IRB application after sending for signatures. Once all signatures have been recorded, you will need to return to this section to submit your application to ORI.

SUBMISSION INFORMATION

0 unresolved comment(s)

Each Section/Subsection in the menu on the left must have a checkmark beside it (except this Submission section) indicating the Section/Subsection has been completed. Otherwise your submission for IRB review and approval cannot be sent to the Office of Research Integrity/IRB.

If applicable, remember to update the Approval Letter Details text box under the Additional Information section

If your materials require review at a convened IRB meeting which you will be asked to attend, it will be scheduled on the next available agenda and you will receive a message to notify you of the date.

If you are making a change to an attachment, you need to delete the attachment, upload a highlighted version that contains the changes (use Document Type of "Highlighted Changes"), and a version that contains the changes without any highlights (use the appropriate Document Type for the item(s)). Do **not** delete approved attachments that are still in use.

Your protocol has been submitted.

Download all

	Document Type	File Loaded	Document Description	File Size	Modified By	Mod Date
	ApprovalLetter	ApprovalLetter.pdf		0.081	klars2	1/8/2024 4:09:24 PM
	Stamped Consent Form	OASIS_PeerP_ElectronicConsent_CLEAN.pdf		0.087	klars2	1/8/2024 4:09:24 PM
	Stamped Consent Form	OASIS_PeerP_PhysicalConsent_Clean.pdf		0.150	klars2	1/8/2024 4:09:24 PM
	Stamped Consent Form	ElectronicConsentStakeholders_ (3).pdf		0.055	klars2	1/8/2024 4:09:23 PM
	Stamped Consent Form	OASIS_PC_Consent.pdf		0.127	klars2	1/8/2024 4:09:23 PM
	Stamped Consent Form	ElectronicConsentPeerCoaches_Clean.pdf		0.064	klars2	1/8/2024 4:09:23 PM
	Stamped Consent Form	OASIS_Stakeholder_Consent.pdf		0.229	klars2	1/8/2024 4:09:23 PM
	ResearchProcedures	Peer Coach Competency Test.docx	Peer Coach Post Training Written Exam	0.955	zmta225	1/5/2024 12:25:44 PM
	ResearchProcedures	PCTraining_01052023.pdf	Peer Coach Training Booklet	8.590	zmta225	1/5/2024 12:23:52 PM
	DataCollection	PFFS_v1.pdf	Pictorial Fit Frail Scale	1.835	zmta225	12/18/2023 2:27:43 PM
	DataCollection	OASIS_Demos .pdf	Demographic and A1c	0.094	zmta225	11/30/2023 11:03:35 AM
	DataCollection	Smoking Measures.pdf	Smoking Habits	0.104	zmta225	10/31/2023 4:34:53 PM
	Advertising	Mailer OASIS .pdf	Mailer for Potential Peer Coaches	0.083	zmta225	10/31/2023 4:30:42 PM
	Informed ConsentParental Permission	OASIS_PeerP_PhysicalConsent_Clean.pdf	Peer Par Physical Consent Clean	0.162	zmta225	10/31/2023 4:10:20 PM
	Informed ConsentParental Permission	OASIS_PeerP_ElectronicConsent_CLEAN.pdf	Peer Par Electronic Consent Clean	0.080	zmta225	10/31/2023 4:09:54 PM
	Informed ConsentParental Permission	OASIS_Stakeholder_Consent.pdf	Stakeholder Consent	0.276	zmta225	10/11/2023 1:20:02 PM
	Informed ConsentParental Permission	ElectronicConsentPeerCoaches_Clean.pdf	REDCap Electronic Consent Peer Coach	0.053	zmta225	10/10/2023 10:23:18 AM
	Informed ConsentParental Permission	OASIS_PC_Consent.pdf	Peer Coach Consent Clean	0.120	zmta225	10/4/2023 1:17:38 PM
	Advertising	Flyers_06162023_Highlighted.pdf	Updated flyers with changes highlighted	1.095	zmta225	6/16/2023 1:49:05 PM
	Advertising	Flyers_06162023.pdf	Updated Flyers	1.088	zmta225	6/16/2023 1:48:47 PM
	Advertising	87024_Advertising_832892.pdf	Stamped flyer with contact info added	1.070	clortz2	5/17/2023 11:33:32 AM
	DataCollection	LUBBEN SOCIAL NETWORK SCALE Short form.pdf	LUBBEN SOCIAL NETWORK SCALE – 6 (LSNS-6)	0.083	clortz2	3/28/2023 3:55:10 PM
	Informed ConsentParental Permission	ElectronicConsentStakeholders_ (3).pdf	E consent redcap stakeholders	0.048	clortz2	3/28/2023 3:52:22 PM
	AddInfoProduct	OASIS DSMP__1 13 23.pdf	DSMP document	0.060	clortz2	2/15/2023 9:05:57 AM
	AddInfoProduct	E-IRB 83904 1st Set of Comments PEER Consent Form.pdf	1st Set of Comments PEER Consent Form	0.273	klars2	1/13/2023 11:34:10 AM

	AddInfoProduct	E-IRB 83904 1st Set of Comments Peer Coach Consent Form.pdf	1st Set of Comments PEER COACH Consent Form	0.266	klars2	1/13/2023 11:14:24 AM
	AddInfoProduct	E-IRB 83904 1st Set of Comments Stakeholder CF.pdf	1st Set of Comments Stakeholders CF	0.245	klars2	1/12/2023 4:04:56 PM
	Advertising	OASIS_Flyers-STAMPED.pdf	Flyers For Recruitment	1.061	clortz2	1/3/2023 11:24:54 AM
	-Letter of Support & Local Context	BSOASISCommunity Action of Southern KY.pdf	Community Action of Southern KY	0.246	clortz2	12/13/2022 1:53:53 PM
	-Letter of Support & Local Context	BSOASISBRADD.pdf	BRADD	0.353	clortz2	12/13/2022 1:53:23 PM
	DataCollection	MOCA-Basic.pdf	MOCA-B	0.482	clortz2	12/13/2022 1:31:24 PM
	ResearchProcedures	Weiner et al., 2017.pdf	Implementation survey	0.634	clortz2	12/13/2022 12:03:34 PM
	ResearchProcedures	Pittman et al., 2021.pdf	PRISM survey	0.932	clortz2	12/13/2022 11:54:47 AM
	ResearchProcedures	PRISM.pdf	PRISM	0.261	clortz2	12/13/2022 11:24:22 AM
	DataCollection	lawton& Brody-iadl.pdf	Lawton"s Instrumental Activities of Daily living	0.080	clortz2	12/12/2022 1:01:47 PM
	DataCollection	Brooks Medication Adherence.pdf	Brooks Medication Adherence Scale	0.573	clortz2	12/12/2022 11:21:37 AM
	DataCollection	Diabetes Self-Management Questionnaire.pdf	Diabetes Self-Management Scale	0.260	clortz2	12/12/2022 11:20:45 AM
	DataCollection	Diabetes Knowledge Questionnaire.pdf	Diabetes Knowledge Questionnaire	0.078	clortz2	12/12/2022 11:20:05 AM
	DataCollection	Problem Areas in Diabetes Survey.pdf	Problem Areas in Diabetes (PAID) Survey	0.518	clortz2	12/12/2022 11:19:22 AM
	DataCollection	Geriatric Depressive Scale Short Form.pdf	Geriatric Depression Scale	0.296	clortz2	12/12/2022 11:18:10 AM
	DataCollection	Medical Outcomes Social Support Survey.pdf	Medical Outcomes Study (MOS) Social Support Survey	0.325	clortz2	12/6/2022 7:54:25 PM
	DataCollection	EuroQofL-5D.pdf	Quality of Life Questionnaire	0.145	clortz2	12/6/2022 7:53:21 PM
	DataCollection	Diabetes Problem-Solving Inventory.pdf	Diabetes Problem Solving Inventory	0.033	clortz2	12/6/2022 7:51:19 PM
	DataCollection	Diabetes Empowerment Scale.pdf	Diabetes Empowerment Scale	0.075	clortz2	11/10/2022 3:16:09 PM

Protocol Changes Protocol Number: 83904

Click link to sort Changed Date
Research Description ResearchProcedures changed by zmta225 on 1/8/2024 3:28:33 PM
There will be two parts to this study that are happening concurrently. Stakeholders will be convening at three timepoints: at study start-up, at study midpoint, and again at the end of the study. While this is happening, peer coaches and peer participants will also be participating in the intervention. See below the procedures each group will follow. ¶
¶
Study Stakeholders ¶
At study start-up, we will recruit study stakeholders (community leaders, older adults with T2D, representatives from BRADD organizations [Aging on Aging case worker, Community Action], etc), obtain informed consent and conduct a series of 1–3 team meetings with them with the following goals: (1) present the final adapted version of the peer health coaching EBI to identify any possible final adaptations to promote pragmatic implementability; (2) finalize operationalization of RE-AIM dimensions based on shared implementation goals; and (3) identify potential facilitators and barriers that might influence implementation. To achieve the third goal, we will use an interview guide tested in previous applications of PRISM (see attachment) across health systems to analyze PRISM contextual determinants, such as external environment, implementation and sustainability infrastructure, and characteristics of participants of the EBI and of the EBI itself. ¶
¶
Midway through the intervention, we will again convene stakeholders for two additional meetings with three primary goals: (1) assess progress on RE-AIM dimensions; (2) select RE-AIM dimensions that require additional attention, if necessary; and (3) give context to understand the reasoning behind any differences that might exist between pre-implementation (i.e., anticipated) and midstream (i.e., unanticipated) adaptations. To achieve the first two goals, we will use an iterative approach to RE-AIM, wherein at the first meeting, team members will be reminded of RE-AIM dimensions selected at project start-up and then asked to confidentially rate the importance and progress of each RE-AIM dimension thus far on a 5-point Likert scale with options to provide qualitative explanatory feedback for each rating. At the second meeting, we will present de-identified results from the survey and then engage in subsequent brainstorming/goal setting to determine best approaches, including possible midcourse EBI adaptations, to improve data collection for RE-AIM dimensions deemed most in need of increased attention. To meet the third goal, we will use a PRISM-based survey (see attached, Pittman et al., 2021) and the same interview guide used at project start-up to give context to determinants of midcourse progress (including any identified adaptations). ¶
¶
Immediately upon program conclusion, we will convene a final team meeting with implementers and research team members, as well as a sample of randomly selected peer health coaches from each of the four factorial conditions, to collect RE-AIM outcomes data and conduct summative interviews with the same PRISM survey and qualitative interview guide as used at project onset and midpoint. Additionally, we will ask research team members, implementers, and selected peer health coaches to complete a psychometrically validated brief 12-item survey (see attached, Weiner et al., 2017) on perceptions of feasibility, acceptability, and appropriateness of both the overall EBI and each of the selected strategies (i.e., each condition in the 2x2 factorial design); we will also randomly select participants from each condition to complete the same survey and complete exit interviews to explore perceived and actual factors underscoring participant satisfaction with the EBI and its components. ¶
¶
The meetings with stakeholders will occur via Zoom or in-person based on availability of members. ¶
¶
Peer Participants ¶
Eligible participants (meeting eligibility criteria and passing screening for no cognitive impairment) will be consented, enrolled and randomized into one of the groups. ¶
1. Self-select mentor, once per week contact. ¶
2. Self-select mentor, every two weeks contact ¶
3. Matched with mentor, once per week contact. ¶
4. Matched with mentor, every two weeks contact. ¶
¶
Participant-peer health coach matching. For participants who are randomized into a group where they are matched with a peer health coach, matching areas include similarity of life stage (e.g., age), county of residence, and duration of disease diagnosis. However, the primary matching area for this study will be county of residence. This decision was informed by key informants who highlighted that Appalachians' sense of identity is closely linked with the county in which they reside and that there will be an additional level of trust and comfort (or lack thereof) based on where their peer resides. ¶
¶
Participant self-selection of peer coach. For participants who are randomized into the group where they are able to self-select their peer coach, they will be provided with profiles of eligible coaches. These profiles will include age, sex, marital status, where they reside (county and town), duration of diabetes diagnosis, current HbA1c, and hobbies. Peer participants will be able to rank their top 3 coaches. The study team will try to match participants with their preferred coach. However, once a coach has been assigned 3 peer participants, they will be removed from the pool of coaches to choose from. ¶
¶
Peer coaches: ¶
Coaches who are interested in the study will contact research team and be prescreened prior to enrollment. As part of the coach screening interviews with the PI, psychosocial status will be assessed described in Data Collection below. The PI will make a final determination of competence, maturity, emotional stability, and verbal communication skills after overall assessment during the screening interview and training. At this time, the research team will obtain consent. Each potential peer coach will undergo pre-intervention training, which is based off of "IDECIDE: Diabetes Peer Coaching Program Toolkit" developed by Dr. Michelle Heisler, and screened by stakeholders for cultural relevance. They will complete structured curriculum modules on working collaboratively with patients, basics of diabetes including self-care activities, knowledge of diabetes medications, recognizing medical "red flags" (e.g., symptoms of hypoglycemia), navigating the clinic, and assessing community resources. The peer coaches will also have training on informal skills development including active listening, non-judgmental communication, and positive social and emotional support (see peer coach training attachment). ¶
Only coaches who pass a written and oral examination are included in the study. Oral examinations will be done by asking participants to preform

mock calls with one another. Study personnel will observe and will score as either pass or fail. ¶

¶

Peer coaches and Peer participants ¶

¶

After peer participants are recruited, consented, and randomly assigned and peer coaches are recruited, consented, and trained, peer participants and coaches will attend an introductory session together, during which the coaching process will be discussed, including time commitment, roles, responsibilities, benefits, and ground rules, and the quads will have the opportunity to ask questions and make informed decisions about their ability to fully participate in the intervention. If face-to-face meetings are not possible for all members of the “quad,” phone or a form of video meeting will be attempted. At this time, the research coordinator will collect demographics, validated questionnaires, and baseline point-of-care clinical outcomes (Hemoglobin A1c via fingerstick). This introductory meeting as well as the peer coach-peer participant meetings during the 6 months of intervention can be in-person in community settings or via Zoom, depending upon participants/coach preference. ¶

¶

Intervention ¶

During the intervention, peer coaches interact with the peer participant for 6 months, with the frequency of interactions based on whichever group the peer participant was randomly assigned to as described above. It is also optional for the peer coach to accompany the participant to at least one clinic visit. Topics to be discussed include current and target clinical goals for A1C, LDL, blood pressure, self-care activities, managing stress, and the SMART objectives. ¶

¶

Coach-peer interactions will be documented with the following information: date, type of encounter (phone, in-person), duration, and topics discussed. ¶

¶

Coaches will attend a monthly meeting to reinforce diabetes knowledge and communication skills. This meeting will be an open discussion based on their experiences interfacing with participants. We will use Zoom for the monthly meetings. ¶

¶

Peer participants will receive a link to a brief REDCap survey by email every two weeks. If internet access is limited, we will send a hardcopy to the participant with a self-addressed stamped envelope. This survey will assess the frequency and duration of calls, other interactions with their peer, and the specific content that was covered. These self-report assessments will be used to track the effectiveness of the intervention. A Modification request can be submitted later for this survey. ¶

¶

At the end of the intervention, the research coordinator will collect HbA1C via fingerstick and the validated questionnaire data described in data collection from peer coaches and peer participants. ¶

¶

Peer participants will then provide HbA1C levels and validated questionnaires again at 3- and 6-months post intervention. ¶

¶

There will be no PHI or health information obtained from or placed into participants' medical records.

Research Description ResearchProcedures changed by zmta225 on 1/5/2024 3:06:15 PM

There will be two parts to this study that are happening concurrently. Stakeholders will be convening at three timepoints: at study start-up, at study midpoint, and again at the end of the study. While this is happening, peer coaches and peer participants will also be participating in the intervention. See below the procedures each group will follow. ¶

¶

Study Stakeholders ¶

At study start-up, we will recruit study stakeholders (community leaders, older adults with T2D, representatives from BRADD organizations [Aging on Aging case worker, Community Action], etc), obtain informed consent and conduct a series of 1–3 team meetings with them with the following goals: (1) present the final adapted version of the peer health coaching EBI to identify any possible final adaptations to promote pragmatic implementability; (2) finalize operationalization of RE-AIM dimensions based on shared implementation goals; and (3) identify potential facilitators and barriers that might influence implementation. To achieve the third goal, we will use an interview guide tested in previous applications of PRISM (see attachment) across health systems to analyze PRISM contextual determinants, such as external environment, implementation and sustainability infrastructure, and characteristics of participants of the EBI and of the EBI itself. ¶

¶

Midway through the intervention, we will again convene stakeholders for two additional meetings with three primary goals: (1) assess progress on RE-AIM dimensions; (2) select RE-AIM dimensions that require additional attention, if necessary; and (3) give context to understand the reasoning behind any differences that might exist between pre-implementation (i.e., anticipated) and midstream (i.e., unanticipated) adaptations. To achieve the first two goals, we will use an iterative approach to RE-AIM, wherein at the first meeting, team members will be reminded of RE-AIM dimensions selected at project start-up and then asked to confidentially rate the importance and progress of each RE-AIM dimension thus far on a 5-point Likert scale with options to provide qualitative explanatory feedback for each rating. At the second meeting, we will present de-identified results from the survey and then engage in subsequent brainstorming/goal setting to determine best approaches, including possible midcourse EBI adaptations, to improve data collection for RE-AIM dimensions deemed most in need of increased attention. To meet the third goal, we will use a PRISM-based survey (see attached, Pittman et al., 2021) and the same interview guide used at project start-up to give context to determinants of midcourse progress (including any identified adaptations). ¶

¶

Immediately upon program conclusion, we will convene a final team meeting with implementers and research team members, as well as a sample of randomly selected peer health coaches from each of the four factorial conditions, to collect RE-AIM outcomes data and conduct summative interviews with the same PRISM survey and qualitative interview guide as used at project onset and midpoint. Additionally, we will ask research team members, implementers, and selected peer health coaches to complete a psychometrically validated brief 12-item survey (see attached, Weiner et al., 2017) on perceptions of feasibility, acceptability, and appropriateness of both the overall EBI and each of the selected strategies (i.e., each condition in the 2x2 factorial design); we will also randomly select participants from each condition to complete the same survey and complete exit interviews to explore perceived and actual factors underscoring participant satisfaction with the EBI and its components. ¶

¶

The meetings with stakeholders will occur via Zoom or in-person based on availability of members. ¶
¶
Peer Participants ¶
Eligible participants (meeting eligibility criteria and passing screening for no cognitive impairment) will be consented, enrolled and randomized into one of the groups. ¶
1. Self-select mentor, once per week contact. ¶
2. Self-select mentor, every two weeks contact ¶
3. Matched with mentor, once per week contact. ¶
4. Matched with mentor, every two weeks contact. ¶
¶
Participant-peer health coach matching. For participants who are randomized into a group where they are matched with a peer health coach, matching areas include similarity of life stage (e.g., age), county of residence, and duration of disease diagnosis. However, the primary matching area for this study will be county of residence. This decision was informed by key informants who highlighted that Appalachians' sense of identity is closely linked with the county in which they reside and that there will be an additional level of trust and comfort (or lack thereof) based on where their peer resides. ¶
¶
Participant self-selection of peer coach. For participants who are randomized into the group where they are able to self-select their peer coach, they will be provided with profiles of eligible coaches. These profiles will include age, sex, marital status, where they reside (county and town), duration of diabetes diagnosis, current HbA1c, and hobbies. Peer participants will be able to rank their top 3 coaches. The study team will try to match participants with their preferred coach. However, once a coach has been assigned 3 peer participants, they will be removed from the pool of coaches to choose from. ¶
¶
Peer coaches: ¶
Coaches who are interested in the study will contact research team and be prescreened prior to enrollment. As part of the coach screening interviews with the PI, psychosocial status will be assessed described in Data Collection below. The PI will make a final determination of competence, maturity, emotional stability, and verbal communication skills after overall assessment during the screening interview and training. At this time, the research team will obtain consent. Each potential peer coach will undergo pre-intervention training, which is based off of "IDECIDE: Diabetes Peer Coaching Program Toolkit" developed by Dr. Michelle Heisler, and screened by stakeholders for cultural relevance. They will complete structured curriculum modules on working collaboratively with patients, basics of diabetes including self-care activities, knowledge of diabetes medications, recognizing medical "red flags" (e.g., symptoms of hypoglycemia), navigating the clinic, and assessing community resources. The peer coaches will also have training on informal skills development including active listening, non-judgmental communication, and positive social and emotional support (see peer coach training attachment). ¶
Only coaches who pass a written and oral examination are included in the study ~~(These exams will be submitted later as a modification request~~).
¶
¶
Peer coaches and Peer participants ¶
¶
After peer participants are recruited, consented, and randomly assigned and peer coaches are recruited, consented, and trained, peer participants and coaches will attend an introductory session together, during which the coaching process will be discussed, including time commitment, roles, responsibilities, benefits, and ground rules, and the quads will have the opportunity to ask questions and make informed decisions about their ability to fully participate in the intervention. If face-to-face meetings are not possible for all members of the "quad," phone or a form of video meeting will be attempted. At this time, the research coordinator will collect demographics, validated questionnaires, and baseline point-of-care clinical outcomes (Hemoglobin A1c via fingerstick). This introductory meeting as well as the peer coach-peer participant meetings during the 6 months of intervention can be in-person in community settings or via Zoom, depending upon participants/coach preference. ¶
¶
Intervention ¶
During the intervention, peer coaches interact with the peer participant for 6 months, with the frequency of interactions based on whichever group the peer participant was randomly assigned to as described above. It is also optional for the peer coach to accompany the participant to at least one clinic visit. Topics to be discussed include current and target clinical goals for A1C, LDL, blood pressure, self-care activities, managing stress, and the SMART objectives. ¶
¶
Coach-peer interactions will be documented with the following information: date, type of encounter (phone, in-person), duration, and topics discussed. ¶
¶
Coaches will attend a monthly meeting to reinforce diabetes knowledge and communication skills. This meeting will be an open discussion based on their experiences interfacing with participants. We will use Zoom for the monthly meetings. ¶
¶
Peer participants will receive a link to a brief REDCap survey by email every two weeks. If internet access is limited, we will send a hardcopy to the participant with a self-addressed stamped envelope. This survey will assess the frequency and duration of calls, other interactions with their peer, and the specific content that was covered. These self-report assessments will be used to track the effectiveness of the intervention. A Modification request can be submitted later for this survey. ¶
¶
At the end of the intervention, the research coordinator will collect HbA1C via fingerstick and the validated questionnaire data described in data collection from peer coaches and peer participants. ¶
¶
Peer participants will then provide HbA1C levels and validated questionnaires again at 3- and 6-months post intervention. ¶
¶
There will be no PHI or health information obtained from or placed into participants' medical records.

Study Personnel Changes:

Modification Comment by Karen Larson - ORI to PI on 1/5/2024 3:31:52 PM
Please upload the Coaches oral exam.

Modification Comment by Karen Larson - ORI to PI on 1/5/2024 2:51:57 PM
Please see if need to revise any sections of RD such as Research Procedures or Data Collection for the Coaches as part of this MR.
