# Supplementary material for: Older adults using social support to improve self-care (OASIS): Adaptation, implementation and feasibility of peer support for older adults with T2D in appalachia: A feasibility study protocol
Source: PLoS One. 2024 Mar 18;19(3):e0300196. doi: 10.1371/journal.pone.0300196 (PMC10947915; doi:10.1371/journal.pone.0300196)
Supplement: S3 File — (PDF) [file pone.0300196.s004.pdf]

University of Kentucky | Office of Research Integrity
IRB, RDRC

XP Initial Review

Approval Ends:	IRB Number:
3/2/2024	83904

TO: Brittany Smalls, PhD
Family and Community Medicine
PI phone #: 859-323-4916

PI email: Brittany.Smalls@uky.edu

FROM: Chairperson/Vice Chairperson
Medical Institutional Review Board (IRB)

SUBJECT: Approval of Protocol

DATE: 3/6/2023

On 3/3/2023, the Medical Institutional Review Board approved your protocol entitled:

Older Adults using Social Support to Improve Self-Care (OASIS): Adaptation, Implementation, and Feasibility of Peer Support for Older Adults with T2DM in Appalachia.

Approval is effective from 3/3/2023 until 3/2/2024 and extends to any consent/assent form, cover letter, and/or phone script. If applicable, the IRB approved consent/assent document(s) to be used when enrolling subjects can be found on the approved application's landing page in E-IRB. [Note, subjects can only be enrolled using consent/assent forms which have a valid "IRB Approval" stamp unless special waiver has been obtained from the IRB.] Prior to the end of this period, you will be sent a Continuation Review (CR)/Annual Administrative Review (AAR) request which must be completed and submitted to the Office of Research Integrity so that the protocol can be reviewed and approved for the next period.

In implementing the research activities, you are responsible for complying with IRB decisions, conditions and requirements. The research procedures should be implemented as approved in the IRB protocol. It is the principal investigator's responsibility to ensure any changes planned for the research are submitted for review and approval by the IRB prior to implementation. Protocol changes made without prior IRB approval to eliminate apparent hazards to the subject(s) should be reported in writing immediately to the IRB. Furthermore, discontinuing a study or completion of a study is considered a change in the protocol's status and therefore the IRB should be promptly notified in writing.

For information describing investigator responsibilities after obtaining IRB approval, download and read the document "PI Guidance to Responsibilities, Qualifications, Records and Documentation of Human Subjects Research" available in the online Office of Research Integrity's IRB Survival Handbook. Additional information regarding IRB review, federal regulations, and institutional policies may be found through ORI's web site. If you have questions, need additional information, or would like a paper copy of the above mentioned document, contact the Office of Research Integrity at 859-257-9428.
